# CD39 regulates P2RX7-mediated lung necrotic lesions in severe experimental tuberculosis

**DOI:** 10.1016/j.mucimm.2026.03.007

**Published:** 2026-03-14

**Authors:** Gislane Almeida-Santos, Igor Santiago-Carvalho, Fabrício Moreira Almeida, Caio César Barbosa Bomfim, Juan Carlo Santos e Silva, Deborah Giovanna Cantarini, Camila Ramos Silva, Martha Simões Ribeiro, Rogério Silva do Nascimento, Bruna de Gois Macedo, Paulo Henrique Lisboa Raeder, Joaquim Teixeira-Xavier, José Maria Alvarez, Mario Hiroyuki Hirata, Robson Coutinho-Silva, Simon C. Robson, Eduardo Pinheiro Amaral, Elena Lasunskaia, Maria Regina D’Império Lima

**Affiliations:** a Universidade de São Paulo (USP), Instituto de Ciêencias Biomeédicas (ICB), Departamento de Imunologia, Saão Paulo, Brazil; b Laboratóorio de Biologia do Reconhecer, State University of North Norte Fluminense Darcy Ribeiro, Campos dos Goytacazes, Rio de Janeiro, Brazil; c Inflammation and Innate Immunity Unit, Laboratory of Clinical Immunology and Microbiology, National Institute of Allergy and Infectious Diseases, National Institutes of Health, Bethesda, MD, USA; d USP, Faculdade de Ciêencias Farmacêeuticas (FCF), Departamento de Anáalises Clínicas e Toxicolóogicas, Sãao Paulo, Brazil; e Instituto de Biofísica Carlos Chagas Filho, Universidade Federal do Rio de Janeiro, Rio de Janeiro, Brazil; f Harvard Medical School, Boston, MA, USA; g Beth Israel Deaconess Medical Center, Boston, MA, USA; h Instituto de Pesquisas Energéeticas e Nucleares (IPEN), Brazil; i University of Virginia School of Medicine, Charlottesville, VA, USA; j Department of Immunology, Mayo Clinic, Scottsdale, AZ, USA; k Institute of Pharmacology and Structural Biology (IPBS), University of Toulouse, CNRS, Toulouse, France

**Keywords:** Tuberculosis, CD39, P2RX7, Necrotic pneumonia, Macrophages

## Abstract

Infection with *Mycobacterium tuberculosis* can cause diverse lesions, such as necrotic pneumonia, which can contribute to tuberculosis progression and transmission between individuals. Despite advances in understanding the role of ATP-gated P2RX7 ion channels in the development of severe forms of the disease, the regulation of this important signaling pathway remains unclear. Herein, we show that the ectonucleotidase CD39 plays an essential regulatory role in tuberculosis progression by preventing lung tissue damage, bacterial dissemination, and excessive inflammatory responses. Mechanistically, through its enzymatic activity on the cellular surface, CD39 protects infected macrophages from undergoing necrotic death mediated by P2RX7 activation. Cell-intrinsic CD39 expression also hinders the establishment of other myeloid cells, such as neutrophils, in the infected lung. We proposed that, by protecting infected macrophages from P2RX7-mediated cell death and bacterial dissemination in the lung tissue, CD39 prevents the development of necrotic lesions. Altogether, these findings uncover a significant role for CD39 as an essential component of the molecular regulation underlying the development of severe tuberculosis.

## Introduction

Tuberculosis (TB), a disease caused by *Mycobacterium tuberculosis* (Mtb), continues to be one of the significant global health challenges, with an estimated 10.7 million cases and 1.23 million deaths in 2025.^1^ The severe pulmonary form of TB is triggered by uncontrolled intracellular bacterial multiplication and, consequently, the necrotic death of infected myeloid cells, leading to extensive tissue necrosis.^2^ Severe lung damage increases the risk of person-to-person transmission, particularly through the formation of cavitary lesions arising from necrotic granulomas, which promote extracellular bacterial multiplication and access to the airways.^3,4^ Therefore, understanding the mechanisms behind the necrotic process and developing strategies to prevent it are vital to controlling the severity of the disease and its global spread.

Macrophage necrosis during Mtb infection is considered a pivotal component in promoting disease progression and is detrimental to the host. Unlike apoptosis, in which the cell contents are retained, necrosis releases intracellular components, including bacteria, into the extracellular milieu. This process not only promotes the dissemination of Mtb within the lung tissue and other organs but also exacerbates inflammation by releasing damage-associated molecular patterns (DAMPs), further contributing to tissue damage and the formation of necrotic granulomas.^4,5^ Necrotic death in Mtb-infected macrophages has been linked to the activation of multiple regulated necrotic death modalities, such as pyroptosis, necroptosis, and ferroptosis, all of each known to drive exacerbation of the host inflammatory response.^6–8^ In addition, studies have shown that macrophages undergoing necrosis fail to effectively control bacterial replication, leading to increased bacterial burden in the lungs and worsening tissue damage.^9,10^ These combined effects are a hallmark of advanced disease, where the immune system fails to eradicate the infection and instead causes irreversible lung damage.^11,12^ Thus, macrophage necrosis in TB represents a detrimental pathway that not only benefits Mtb survival but also drives pathological tissue destruction.

Our previous study has shown that sustained P2RX7 signaling, intensified by high levels of extracellular ATP (eATP), detrimentally affects the progression of TB in mice,^13^ particularly in cases involving a hyperinflammatory condition induced by hypervirulent mycobacterial strains.^14^ This evidence suggests that the eATP-P2RX7 axis exacerbates tissue damage under intense cellular stress triggered by the infection. Consistent with this idea, inhibition of P2RX7 during the acute phase of TB has been shown to improve disease outcome, as evidenced by optimal activation of host immune defenses against Mtb, non-necrotic granulomatous pneumonia, and reduced pathogen dissemination from the lung to other organs.^15^

Interestingly, the enzymatic activity of CD39 in degrading eATP has been proposed as an essential regulatory mechanism of the immune response during infection and cancer.^16–18^ In this context, CD39 hydrolyzes ATP to adenosine diphosphate (ADP) and adenosine monophosphate (AMP), which further degrade to adenosine.^19^ Previous studies have shown that CD39 exhibits a protective effect on preventing liver necrosis by negatively regulating P2RX7-mediated cell death.^20^ CD39 expression on regulatory T cells and CD4^+^ T cells has been shown to impair macrophage activation and bacterial clearance during TB, contributing to immune evasion.^21,22^ Moreover, purinergic signaling via CD39 has been implicated in modulating the balance between pro- and anti-inflammatory responses during Mtb infection, thereby altering disease progression.^23^ Although these findings suggest a potential role for CD39 in TB pathogenesis, its direct involvement in regulating macrophage cell death and host immune responses during Mtb infection remains unclear. In this study, we uncovered the effects of CD39 activity on lung immunopathology and its robust association with necrotic lesions during TB. Our data strongly indicate that CD39 expression on immune cells, particularly macrophages, reduces lung necrosis and bacterial dissemination, and enhances survival in mice. This underscores the potential of targeting eATP signaling to diminish the widespread necrotic cell death and exacerbated host inflammatory response that worsens TB progression.

## Results

### The expression of genes encoding CD39 and P2RX7 is highly correlated with active forms of TB in mice and human patients

**Fig. 1.**
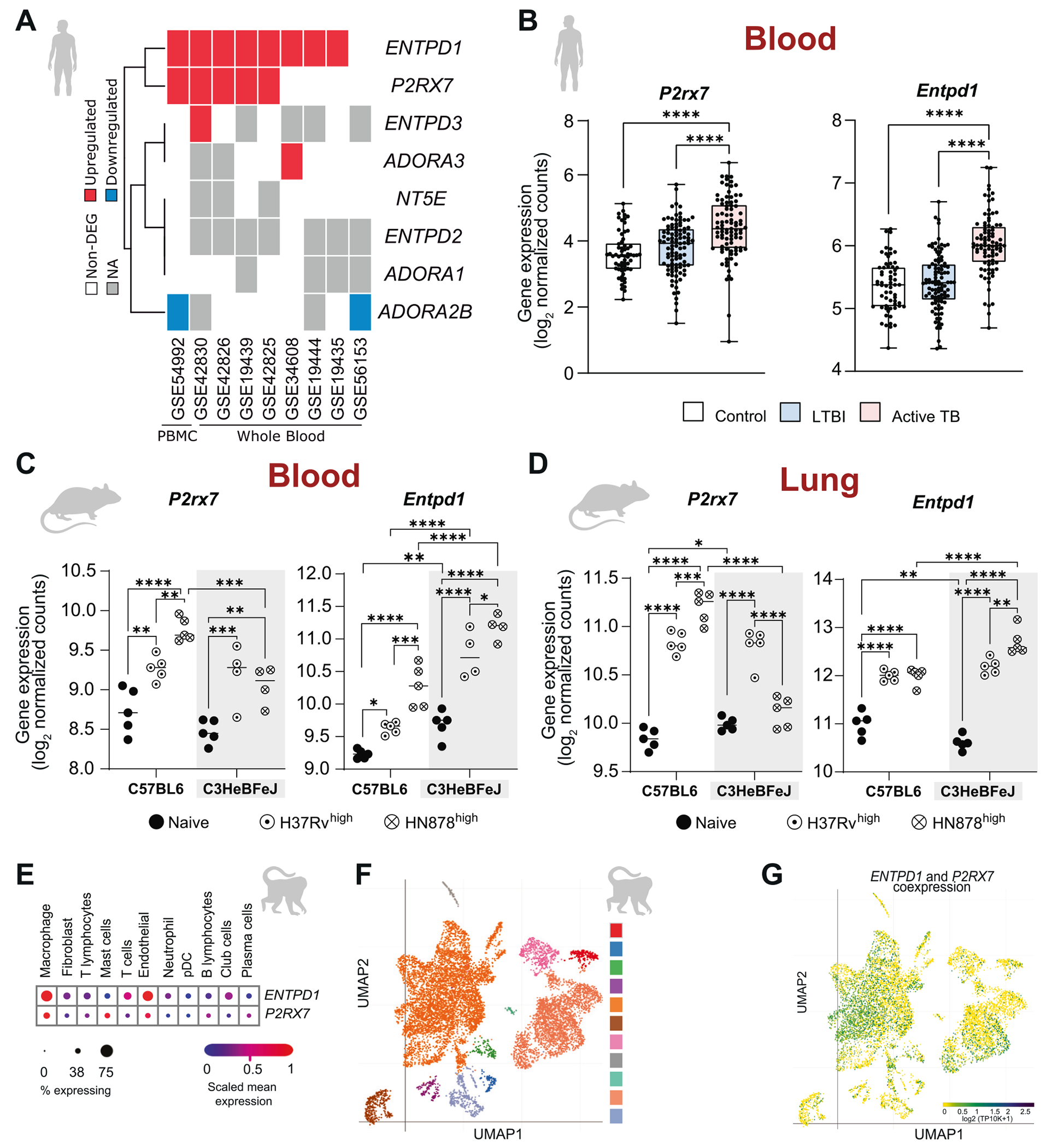
The expression of genes encoding CD39 and P2RX7 is highly correlated with active forms of TB in human patients, mice, and non-human primates. (A) Expression levels of purinergic genes (*ENTPD1*-CD39, *P2RX7*-P2RX7, *ENTPD2-3*, *NT5E*, and *ADORA1,2b,3*) in whole and peripheral blood samples from patients with active TB were analyzed using publicly available datasets. GEO from each dataset is displayed. (B) Comparative analysis of *ENTPD1* and *P2RX7* gene expressions in blood samples from patients with active TB, latent TB infection (LTBI), and healthy controls derived from publicly available RNA-seq datasets. (C and D) Transcriptional levels of *Entpd1* and *P2rx7* in the blood and lung of C57BL/6 and C3HeB/FeJ mice infected with a high dose of *Mycobacterium tuberculosis* H37Rv and HN878 strains compared to non-infected (naïve) mice, using publicly available RNA-seq datasets. (E) Cell Type Annotation analysis in granulomas from non-human primates, based on public high-throughput single-cell mRNA sequencing datasets. (F and G) UMAP plots show the distribution of immune cell populations and the coexpression of *P2RX7* and *ENTPD1* across these populations in granulomas from non-human primates. Abbreviations: LTBI-latent TB infection, PBMC-peripheral blood mononuclear cells. Normalization and statistical procedures for each panel are detailed in Methods. Dataset sources and full metadata are provided in Supplementary Table S1. Significant differences between groups are indicated by p < 0.05 (*), p < 0.01 (**), p < 0.001 (***), and p < 0.0001 (****), as determined by the Mann-Whitney non-parametric test or one-way ANOVA with Tukey post hoc test.

To evaluate purinergic gene expression during Mtb infection, we analyzed publicly available datasets of whole and peripheral blood samples from patients with active TB.^24–26^ Dataset sources and term definitions are provided in Supplementary Table S1. Meta-analysis of *ENTPD1* (CD39) gene expression was performed, revealing a strong upregulation of the gene compared to healthy subjects in all eight independent reports evaluated (Fig. 1A). Furthermore, enhanced expression of the *P2RX7* gene was found in five of these reports, demonstrating a robust correlation between these two genes during Mtb infection. We failed to detect differences in the expression of other genes in the purinergic pathway (ENTPD2-3, NT5E, and ADORA1,2b,3) in patients with active TB. To determine whether this response depends on disease activity, we also analyzed a publicly available RNA-seq dataset.^26–28^ *ENTPD1* and *P2RX7* expression were found to be increased in the blood of active TB patients compared to healthy controls and subjects with latent TB infection (LTBI) (Fig. 1B). No significant difference was observed between LTBI and healthy controls, suggesting that *ENTPD1* and *P2RX7* upregulation is associated with active forms of the disease.

To determine whether the transcriptional signature observed in humans is recapitulated in mouse models and whether *Entpd1* and *P2rx7* gene expression associates with disease severity across host and pathogen contexts, we analyzed publicly available datasets from the blood and lungs of resistant C57BL/6 and necrosis-prone C3HeB/FeJ mice infected with either the virulent laboratory strain H37Rv or the hypervirulent strain HN878 at high dose, infection conditions associated with the development of necrotic lung pathology in the original study.^29^ The transcriptional mRNA levels of *Entpd1* and *P2rx7* were elevated in both blood and bulk lung tissue from Mtb-infected mice compared to naïve animals, as observed in human disease (Fig. 1C and D). In blood, infection with either strain induced a significantly greater increase in *Entpd1* expression in C3HeB/FeJ mice compared to C57BL/6 mice, whereas *P2rx7* did not show a parallel strain-dependent difference. In bulk lung tissue, *Entpd1* transcriptional levels were higher in the susceptible C3HeB/FeJ strain compared to C57BL/6 and were further increased following infection with the hypervirulent HN878 strain, whereas no difference between Mtb strains was observed in resistant C57BL/6 mice. In contrast, bulk lung *P2rx7* transcriptional mRNA levels increased in C57BL/6 mice infected with HN878 but were reduced in infected C3HeB/FeJ mice, which may reflect reduced immune cell–associated transcriptional signal in extensively necrotic lesions, as reported in the original study. Together, these findings support that *Entpd1*/CD39 transcription associates with necrosis-prone disease phenotypes across host susceptibility and pathogen virulence contexts.

To provide an overview of genes encoding CD39 and P2RX7 across different cell subtypes in the infected lung, we analyzed a public high-throughput single-cell mRNA sequencing dataset of granulomas from non-human primates (NHP) at 4 weeks p.i.^29^ A Cell Type Annotation analysis showed a higher mRNA expression of *ENTPD1* on endothelial cells and macrophages than on other immune and non-immune cells (Fig. 1E). Interestingly, *P2RX7* expression was slightly increased on endothelial cells, macrophages, and mast cells within the granuloma (Fig. 1E). Importantly, among all cell subtypes, macrophages were the most abundant population (Fig. 1F) and the cell type showing the highest *ENTPD1* and *P2RX7* coexpression (Fig. 1G).

### CD39 deficiency aggravates pulmonary necrosis caused by hypervirulent mycobacteria

**Fig. 2.**
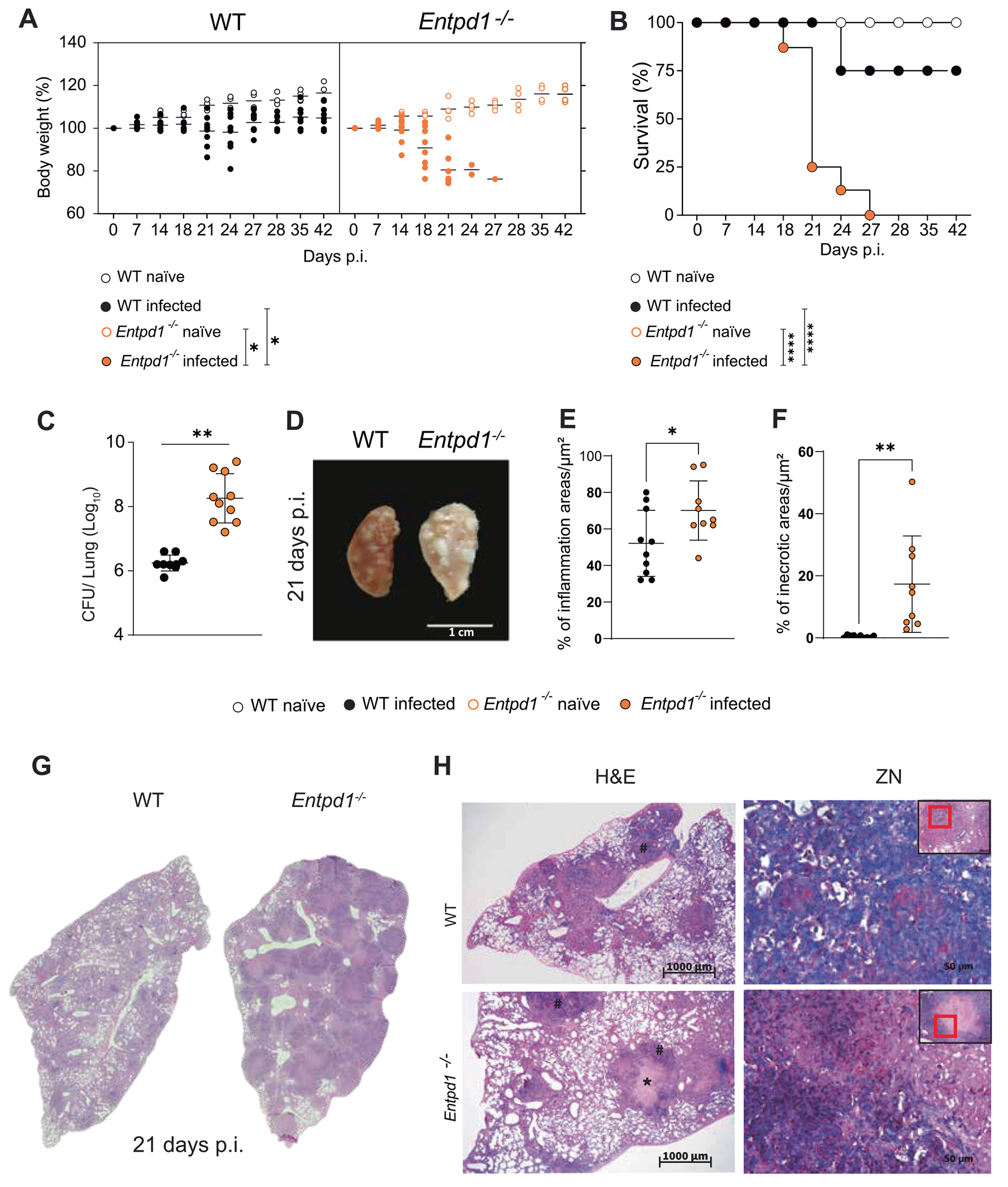
CD39 deficiency aggravates pulmonary necrosis caused by hypervirulent mycobacteria. C57BL/6 wild-type (WT) and CD39-deficient (*Entpd1*^−/−^ ) mice were infected with the hypervirulent M299 Mtb strain. Non-infected (naïve) mice were used as controls. Lungs were analyzed at day 21 of infection. (A) Percent change in body weight from baseline (day 0). (B) Survival, with humane endpoint at >20% weight loss. (C) Lung bacterial burden (CFU). (D) Gross pathology of the left lung. (E–F) Blinded quantitative histomorphometry of H&E sections showing % inflammatory area (E) and % necrotic area (F) per section. (G) Representative scans the whole left lung lobe in the respective infected WT and *Entpd1*^−/−^ . (H) H&E and Ziehl-Neelsen (ZN) stained lung sections. Necrotic spots are marked with an asterisk (*), and areas of alveolitis are indicated by a hash symbol (#). Significant differences between groups are indicated by p < 0.05 (*), and p < 0.01 (**), as determined by the Mann-Whitney non-parametric test or one-way ANOVA with Tukey post hoc test. The data represent results from two independent experiments, with five mice per group.

We previously defined that P2RX7 signaling promotes the development of necrotic lesions in mice infected with highly virulent mycobacteria.^13^ To investigate the role of CD39 in severe TB, we infected *Entpd1*^−/−^ and their WT littermates with the highly virulent Beijing M299 Mtb clinical isolate, which triggers necrotic lung pathology in WT mice.^13,30^ *Entpd1*^−/−^ mice had anticipated and accentuated weight loss compared to WT mice (Fig. 2A). Additionally, all *Entpd1*^−/−^ mice died within 18–27 days p.i. (Fig. 2B). At the same time, 87.5% of the WT mice remained alive during a 42-day p.i. period. Lung collected 21 days p.i. showed a 200-fold increase in the bacterial burden in *Entpd1*^−/−^ mice compared to WT mice (Fig. 2C). Notably, increased presence of lung white nodules was observed in *Entpd1*^−/−^ mice compared to WT mice (Fig. 2D). Lung tissue damage was then quantified by blinded histomorphometry that showed higher percentages of inflammatory and necrotic areas in *Entpd1*^−/−^ mice compared with WT mice (Fig. 2E and F), consistent with extensive necrotic pneumonia, alveolitis, and edema observed in representative images (Fig. 2G and H). These results suggest that CD39 expression suppresses the development of severe lung disease, as evidenced by increased necrotic pneumonia and mortality in *Entpd1*^−/−^ mice infected with highly virulent Mtb.

### The expression of CD39 in the immune cells protects against severe TB

**Fig. 3.**
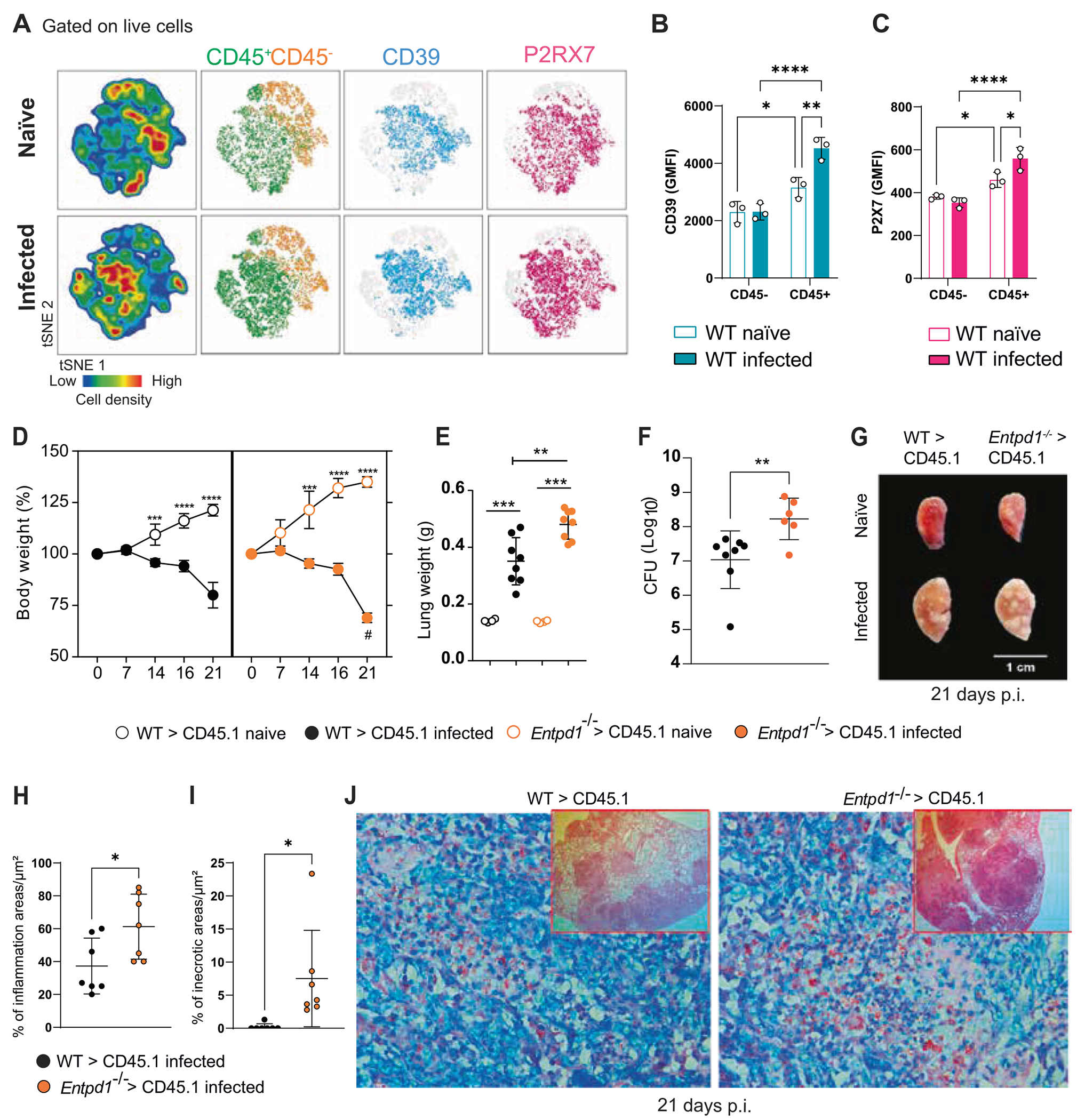
The expression of CD39 in the immune cells protects against severe TB. C57BL/6 (WT) mice, as well as WT>CD45.1 (WT) and *Entpd1*^−/−^ >CD45.1 bone marrow mouse chimeras, were infected with the M299 Mtb strain. Non-infected (naïve) mice were used as controls. Lungs were analyzed at day 21 of infection. (A) Distribution of immune (CD45^+^) and non-immune (CD45^−^) cells in the lungs of naïve and infected WT mice. (B and C) Geometric Median intensity fluorescence (GMFI) of CD39 and P2RX7 expression in CD45^+^ and CD45^−^ lung cells is described in A. (D) Percentage changes in body weight relative to baseline (day 0) in infected mouse chimeras. (E and F) Lung weights and colony-forming units (CFUs) in lung homogenates of infected mouse chimeras. (G) Gross macroscopic pathology of the left lung in mouse chimeras. (H) Blinded quantitative histomorphometry of H&E sections showing percentages of inflammation and necrotic areas. (I) Histopathological analysis with Ziehl-Neelsen staining of lung sections of mouse chimeras. Significant differences between groups are indicated by p < 0.05 (*), p < 0.01 (**), p < 0.001 (***), and p < 0.0001 (****), as determined by the Mann-Whitney non-parametric test or one-way ANOVA with Tukey post hoc test. The data represent results from two independent experiments, five mice per group.

To determine whether the protective effect of CD39 activity was associated with its expression on immune or epithelial cells, we next evaluated the protein levels of CD39 and P2RX7 in immune (CD45^+^) and non-immune (CD45^−^) lung compartments of WT mice following M299 Mtb infection. The results revealed the accumulation of immune cells in the infected lungs (Fig. 3A), with increased proportions and expression of CD39 and P2RX7 compared with non-immune cells (Fig. 3B and C).

Next, we evaluated whether CD39 expression in the immune compartment was required for protection against severe TB using WT>CD45.1 (WT) and *Entpd1*^−/−^ >*CD45.1* bone marrow chimeras. *Entpd1*^−/−^ >*CD45.1* mice were more susceptible to infection and showed accentuated body weight loss at day 21p.i. compared to WT>*CD45.1* (Fig. 3D). The lung weight and bacterial burden in *Entpd1*^−/−^ >*CD45.1* mice were also increased compared to WT>*CD45.1* mice (Fig. 3E and F). Enlarged lung white nodules were found in infected *Entpd1*^−/−^ >*CD45.1* mice (Fig. 3G), which showed larger inflammatory and necrotic areas with extracellular bacteria compared to *WT>CD45.1* mice (Fig. 3H–J). Because reciprocal chimeras (CD45.1>WT and CD45.1>*Entpd1*^−/−^ ) were not performed, we concluded that CD39 deficiency in hematopoietic cells is sufficient to exacerbate disease, without excluding a role for structural cells.

### Ablation of CD39 results in lower numbers of macrophages and dendritic cells while increasing the inflammatory response in infected lungs

**Fig. 4.**
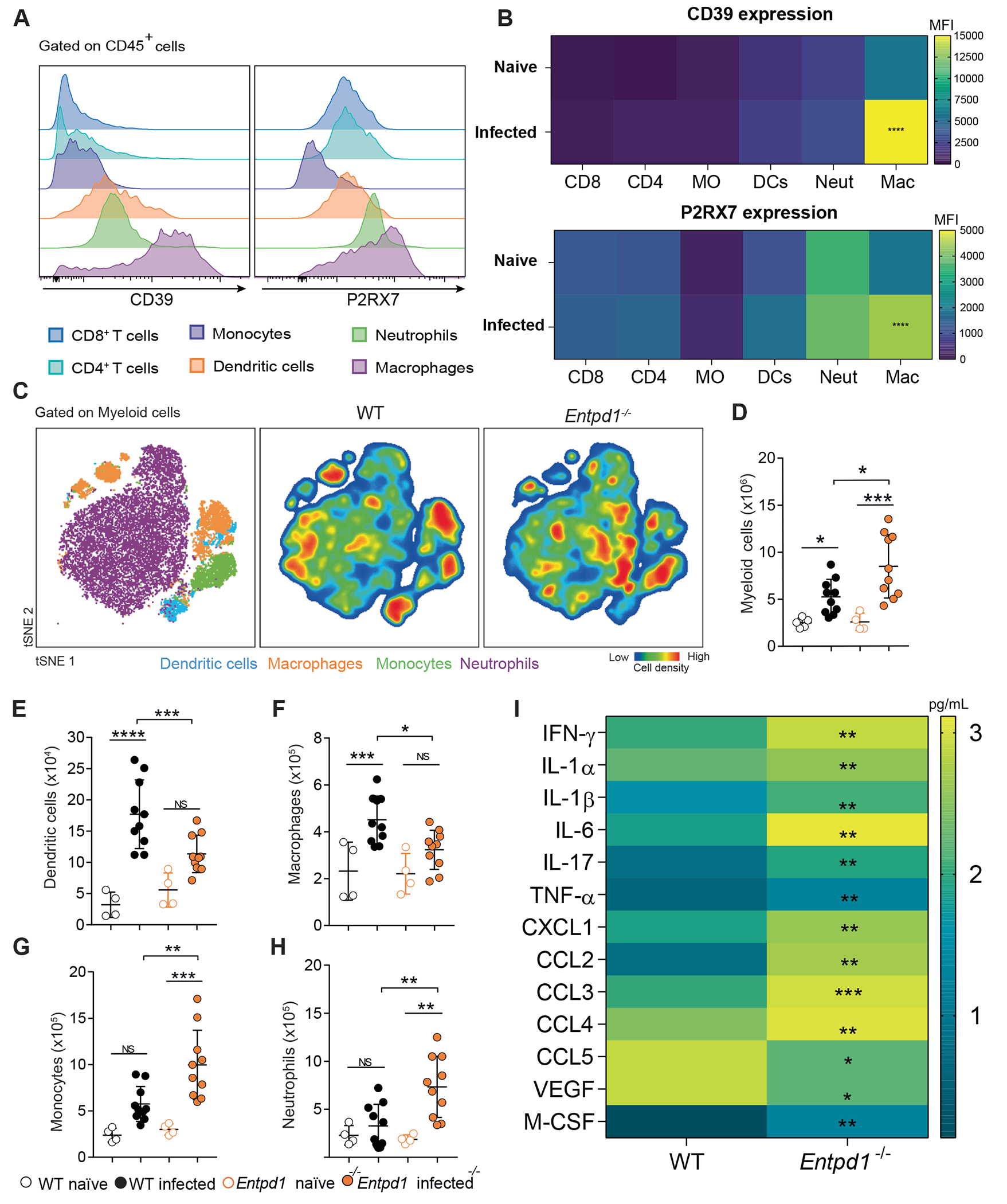
Ablation of CD39 results in lower numbers of macrophages and dendritic cells while increasing the inflammatory response in infected lungs. C57BL/6 wild-type (WT) and CD39-deficient (*Entpd1*^−/−^ ) mice were infected with the hypervirulent M299 Mtb strain. Non-infected (naïve) mice were used as controls. Lungs were analyzed on day 21 of infection. (A) Histograms showing CD39 and P2RX7 protein expression in immune cell subsets of infected WT mice (gate strategy in Supplementary Fig. S1A). (B) Heatmap illustrates the Geometric Mean Fluorescence Intensity (GMFI) of P2RX7 and CD39 in immune cell subsets of naïve and infected WT lungs (gate strategy and FMO controls in Supplementary Fig. S1A and B). (C) t-SNE plots depicting the distribution of concatenated myeloid cells from infected WT and *Entpd1*^−/−^ mice (gate strategy in Supplementary Fig. S3). (D–H) Absolute numbers of myeloid cells, dendritic cells, macrophages, monocytes, and neutrophils in naïve and infected WT and *Entpd1*^−/−^ mice (gate strategy in Fig. S3). (I) Cytokine and chemokine levels (pg/mL) in lung homogenates of infected WT and *Entpd1*^−/−^ mice. Significant differences between groups are indicated by p < 0.05. Significance levels are represented as follows: p < 0.05 (*), p < 0.01 (**), p < 0.001 (***), and p < 0.0001 (****), determined by the Mann-Whitney non-parametric test or one-way ANOVA with Tukey post hoc test. The data represent results from two independent experiments with five mice per group.

To investigate which immune cell population is primarily responsible for CD39’s protective role in preventing TB severity, we evaluated CD39 and P2RX7 expression in macrophages, neutrophils, monocytes, dendritic cells, and T cells from the lungs of infected WT mice. Gate strategy and FMO controls for the expression of CD39 and P2RX7 on each immune cell population are represented in Fig. S1. Our antibody panel identified CD11c^+^MHCII^+^ myeloid antigen-presenting cells but lacked markers to unambiguously resolve dendritic-cell subsets under inflammatory conditions, nor did it discriminate between alveolar and interstitial macrophages. We found that macrophages exhibited higher expression of CD39 and P2RX7 and were the only immune cell population with increased levels of both molecules in infected WT mice compared to naïve counterparts (Fig. 4A and B, gate strategy in Fig. S1A and B). These results parallel RNA transcription levels observed in the single-cell analysis of granulomas from non-human primates (Fig. 1E–G).

Next, we investigated the impact of CD39 deficiency on the inflammatory response in WT and *Entpd1*^−/−^ infected lungs. We previously demonstrated that the excessive CD4^+^ T cell response in the lung parenchyma is detrimental to the host in TB caused by highly virulent mycobacterial strains, exacerbating pneumonia and consequent disease severity.^31^ Although there was an increase in lung CD4^+^ T cell population in *Entpd1*^−/−^ mice compared to WT mice, no difference in the numbers of parenchymal CD44^+^CD4^+^ T (CD45 i.v.^−^) or intravascular (CD45 i.v.^+^) CD44^+^CD4^+^ T or CD44^−^ CD4^+^ T cells was observed (Fig. S2A–D). CD69 expression was slightly increased in parenchymal CD44^+^CD4^+^ T cells from *Entpd1*^−/−^ mice (Fig. S2E, gate strategy in Fig. S2F). In contrast, the t-SNE plots overlaid with gating from the conventional analysis provided an illustrative visualization of changes in the myeloid cell compartment, showing a proportional reduction in macrophage and dendritic cell populations in *Entpd1*^−/−^ infected lungs (Fig. 4C). This finding was confirmed by analyses of the numbers of each cell population per lung using the conventional gating hierarchy (Fig. 4D–H, gate strategy in Fig. S3). Despite an increase in the absolute number of myeloid cells per lung in infected *Entpd1*^−/−^ mice (Fig. 4D), the absolute numbers of macrophages and dendritic cells per lung were reduced compared with infected WT mice (Fig. 4E and F). At the same time, neutrophil and monocyte populations augmented in the absence of CD39 (Fig. 4G and H). In addition, we observed increased levels of cytokines implicated in the necrotic process (IL-1α and IL-1β), cell migration (CCL2, CCL3, and CCL4), and effector T cell response (IFN-γ, TNF-α, IL-17) in lung homogenates of infected *Entpd1*^−/−^ mice (Fig. 4I). These findings suggest that the lack of CD39 leads to the depletion of macrophages and dendritic cells while promoting the inflammatory response against TB.

### CD39 deficiency leads to an increased myeloid cell death and caspase-1 activation in Mtb-infected Entpd1^−/−^ mice

**Fig. 5.**
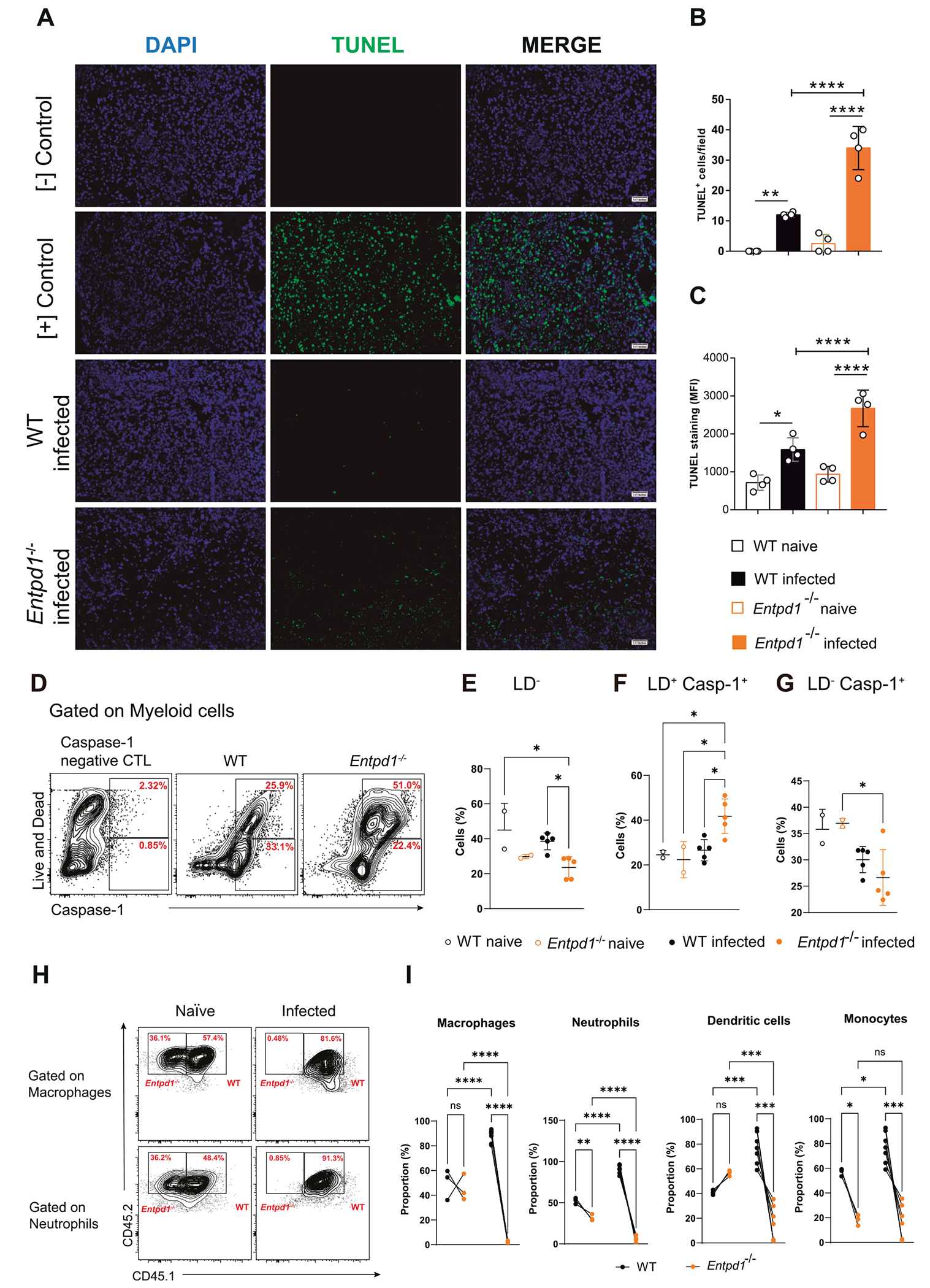
CD39 deficiency leads to an increased myeloid cell death and caspase-1 activation in Mtb-infected *Entpd1*^−/−^ mice. (A-G) C57BL/6 wild-type (WT) and CD39-deficient (*Entpd1*^−/−^ ) mice were infected with the hypervirulent M299 Mtb strain. Non-infected (naïve) mice were used as controls. Lungs were analyzed at day 21 of infection. (A) TUNEL staining in lung histological sections. (B) Number of TUNEL^+^ cells/field in histological sections described in A. (C) Mean Fluorescence Intensity (MFI) of TUNEL staining in histological sections described in A. Each dot represents the mean of six fields per mouse. (D) Flow cytometry dot plots of viability staining (Live and Dead) and active caspase-1 staining in myeloid (Live/Dead^−^ , DUMP^−^ , CD45^+^) lung cells. (E–G) Percentages of live cells, live active caspase 1^+^ cells, and dead active caspase 1^+^ cells in myeloid cells. (H–I) WT (45.1/2) and *Entpd1*^−/−^ (45.2) (1:1 ratio) bone marrow chimeras were infected with the hypervirulent M299 Mtb strain. Lungs were analyzed at day 21 of infection. (H) Flow cytometry dot plots of macrophages and neutrophils. (I) Proportions of WT and *Entpd1*^−/−^ macrophages, neutrophils, dendritic cells, and monocytes in infected lungs. The data represent two independent experiments. Significant differences between groups are indicated by p < 0.05. Significance levels are described as follows: p < 0.05 (*), p < 0.01 (**), p < 0.001 (***), and p < 0.0001 (****), determined by the Mann-Whitney non-parametric test or one-way ANOVA with Tukey post hoc test.

To assess whether the low macrophage numbers and high IL-1β levels in the lungs of infected *Entpd1*^−/−^ mice were associated with increased cell death, we stained lung histological sections using the TUNEL method. Considering both the number of positive cells and the mean fluorescence intensity (GMFI), a higher level of DNA damage was observed in the lungs of infected *Entpd1*^−/−^ mice compared to infected WT mice (Fig. 5A–C).

We next investigated whether increased DNA fragmentation in the lungs of infected *Entpd1*^−/−^ mice was associated with myeloid cell death by assessing cell viability and caspase-1 activation. A percentage reduction of live cells (LD^−^ ) and an increase of active caspase-1^+^ dead cells (LD^+^ Casp-1^+^) were observed in infected *Entpd1*^−/−^ mice compared to infected WT mice (Fig. 5D–F), while there was no difference in the active caspase-1^+^ live population (LD^−^ Casp-1^+^) (Fig. 5G). Of note, a population of dead cells expressing high levels of active caspase-1 became evident only in the absence of CD39. These results indicate that CD39 expression attenuates inflammasome activation and myeloid cell death, possibly by a mechanism dependent on pyroptosis.

Macrophage cell death and increased neutrophil infiltration are common endpoints of severe necrotic lesions in the lungs of Mtb-infected mice, creating a niche for bacterial dissemination.^30,32,33^ We hypothesize that increased myeloid cell death in infected *Entpd1*^−/−^ lungs is related to increased susceptibility of macrophages and neutrophils to cell death. To test this hypothesis *in vivo*, we developed mixed bone marrow chimeras reconstituted with WT and *Entpd1*^−/−^ bone marrow cells (1:1 ratio) and compared myeloid cell population frequencies on day 21 of infection. The frequencies of neutrophils and monocytes were altered from baseline levels, whereas the frequencies of macrophages and dendritic cells were not different. After infection, the discrepancies became more evident, with a much higher frequency of WT macrophages, neutrophils, dendritic cells, and monocytes in the lungs of infected mice (Fig. 5H and I). These findings show that in a competitive setting, *Entpd1*^−/−^ myeloid cells have impaired ability to establish in the infected lungs compared to WT myeloid cells, and that CD39 expression is also essential for their survival or establishment in the lungs following infection.

### CD39 deficiency induces Mtb-infected macrophage death and extracellular bacterial release, which is rescued by P2RX7 inhibition

**Fig. 6.**
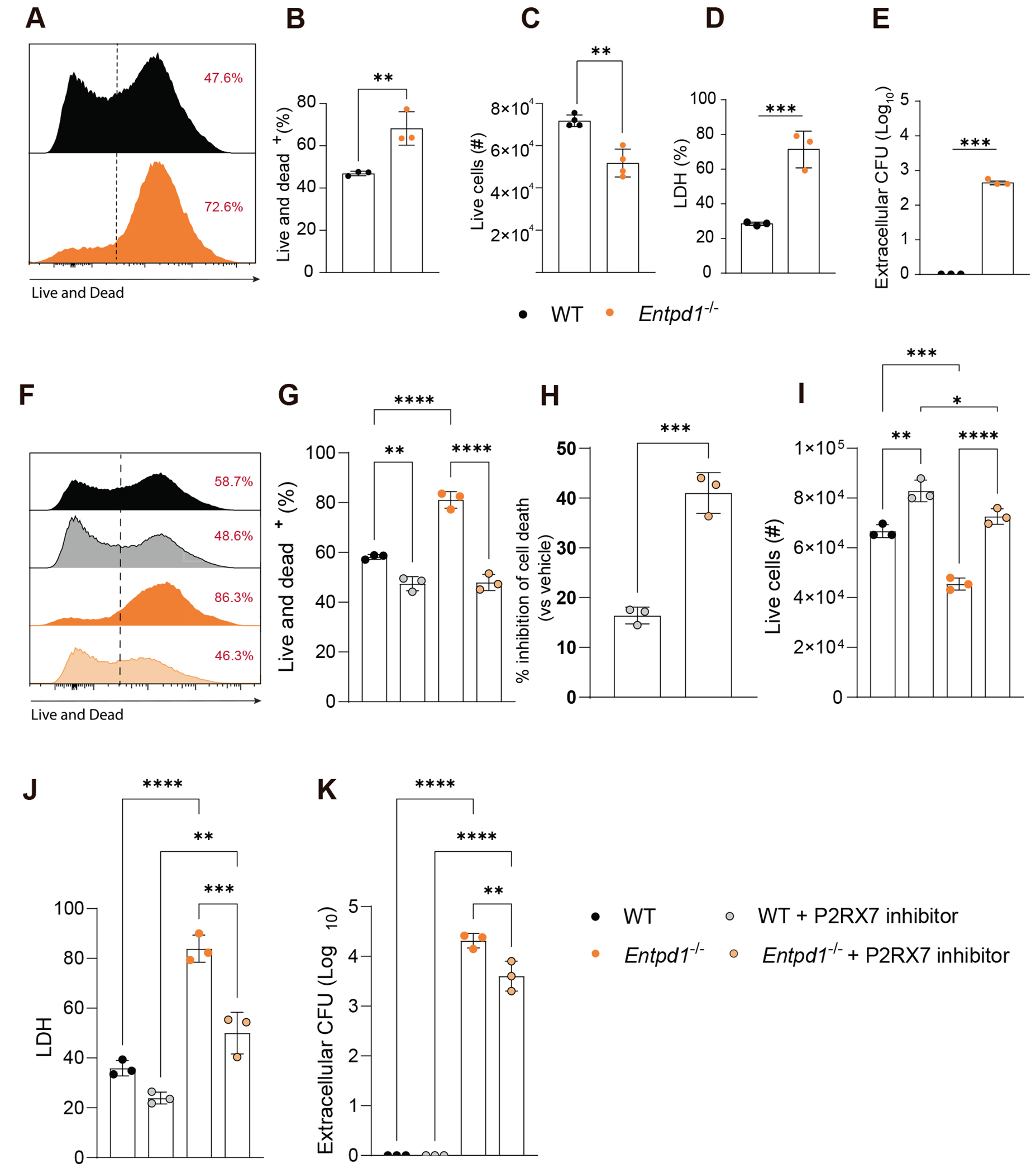
CD39 deficiency induces Mtb-infected macrophage death and extracellular bacterial release, which is rescued by P2RX7 inhibition. (A) Histograms of viability staining (Live and Dead) in bone marrow-derived macrophages (BMDM) from WT or *Entpd1*^−/−^ mice at 24 h p.i. *in vitro* with a MOI of 10. (B and C) Percentage of dead cells and absolute numbers of live cells per well in BMDM cultures described in A. (D and E) Extracellular CFU and percentages of LHD release in the supernatant of infected BMDM cultures are described in A. (F) Histograms showing viability staining (Live and Dead) of bone marrow-derived macrophages (BMDMs) from *Entpd1*^−/−^ mice treated or not with the P2RX7 inhibitor A740003. Cells were maintained in culture for 24 h p.i. with a MOI of 10. (G) Frequencies of live cells in cultures described in F. (H). For clarity, we also summarize the inhibitor effect as percent inhibition of cell death relative to paired vehicle controls. Percentage of cell death inhibition of treated cells compared to their vehicle-treated controls. (I) Absolute numbers of BMDMs in cultures. (J and K) Extracellular CFU and percentage of LHD release in the supernatant of infected BMDM cultures. The data represent two independent experiments. Significant differences between groups are indicated by p < 0.05. Significance levels are represented as follows: p < 0.05 (*), p < 0.01 (**), p < 0.001 (***), and p < 0.0001 (****), determined by the Mann-Whitney non-parametric test or one-way ANOVA with Tukey post hoc test. The data represent two independent experiments with three to four mice per group.

Because macrophages are the early Mtb niche and macrophage cell death triggers bacillary spread and lung necrosis,^34,35^ we infected WT and *Entpd1*^−/−^ bone marrow-derived macrophages (BMDMs) with Mtb M299 at an MOI of 10 to test whether CD39 deficiency increases susceptibility to Mtb-induced cell death. Cell viability, live cell counts, LDH release, and extracellular CFU in supernatants were evaluated after 24 h of culture. CD39 ablation resulted in 25% more dead (Live and Dead^+^) BMDMs following infection (Fig. 6A and B) and a significant reduction in the absolute numbers of these cells (Fig. 6C). Culture supernatants from these cells showed higher levels of lactate dehydrogenase (LDH), a marker of membrane integrity loss, and increased numbers of extracellular mycobacteria suggesting that infected *Entpd1*^−/−^ BMDMs were more susceptible to cell lysis (Fig. 6D and E). These data suggest that CD39 prevents the death of infected macrophages, thereby limiting extracellular bacterial spread.

It is known that CD39 can regulate macrophage death and the release of pro-inflammatory cytokines induced by P2RX7 signaling.^36^ Therefore, we next investigated whether the protective effect of CD39 on Mtb-infected killing resulted from its ability to degrade eATP and prevent P2RX7 signaling. During infection with Mtb M299 (MOI of 10), pharmacological blockade of P2RX7 with A740003 reduced the mortality of WT and *Entpd1*^−/−^ BMDMs (Fig. 6F and G). Remarkably, this inhibitory effect was more pronounced in *Entpd1*^−/−^ BMDMs than in WT BMDMs (Fig. 6H), resulting in comparable percentages of dead cells independently of CD39 expression (Fig. 6H). A comparable effect was observed for the absolute number of live cells per culture (Fig. 6I) and LDH release (Fig. 6J), as well as a 5–10 times reduction in the number of extracellular CFUs in supernatants (Fig. 6K). These data indicate that CD39 restrains eATP-P2RX7-driven cell lysis in infected macrophages and extracellular bacterial release, while the measurable benefit of its inhibition in infected WT BMDMs shows that P2RX7 can be engaged even when CD39 is present.

### In vivo P2RX7 inhibition limits necrotic pathology and bacterial expansion while preserving the macrophage population

**Fig. 7.**
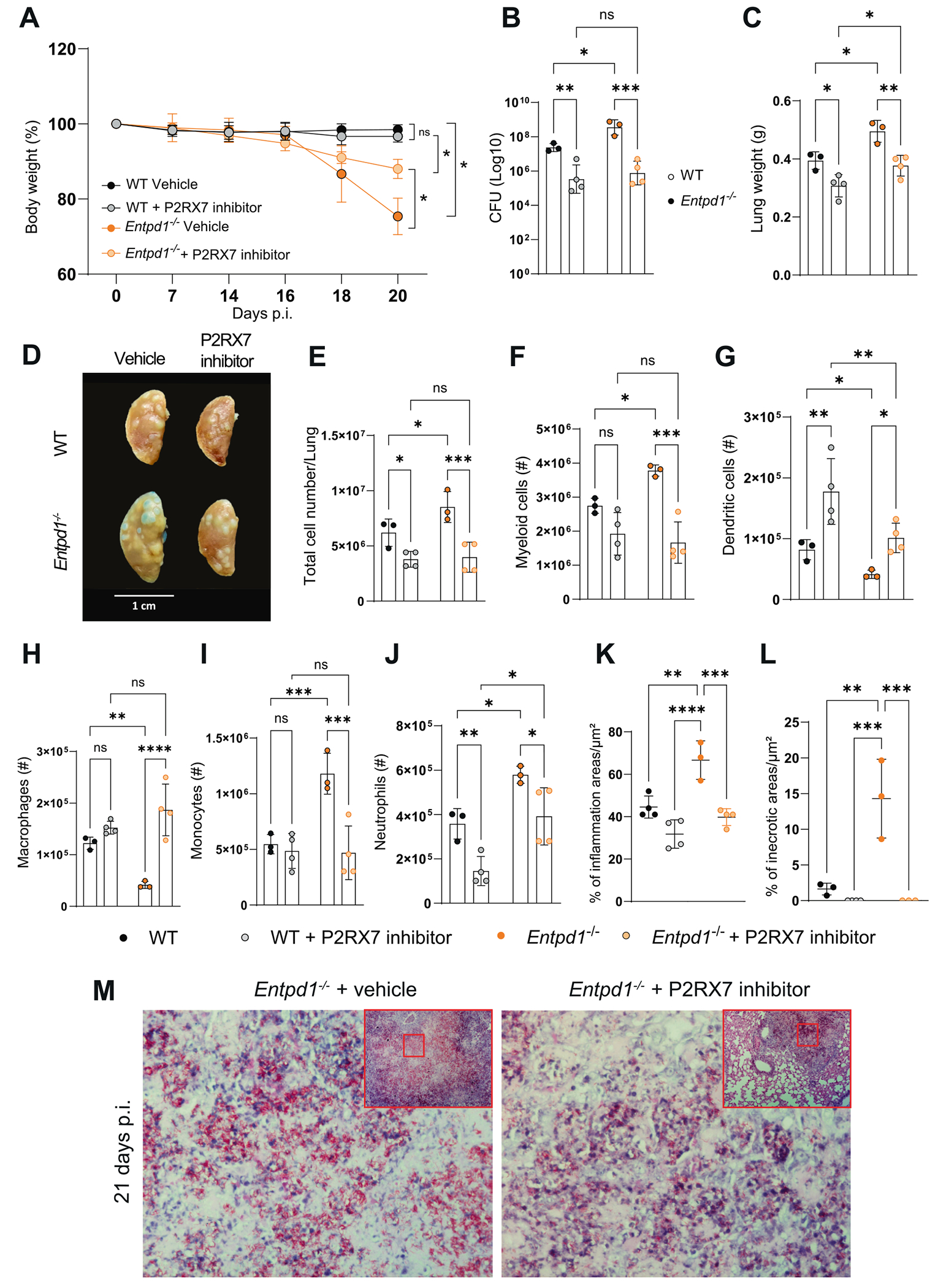
*In vivo* P2RX7 inhibition limits necrotic pathology and bacterial expansion while preserving the macrophage population. WT and *Entpd1*^−/−^ mice infected with the hypervirulent M299 Mtb strain were treated or not with BBG from 14 to 20 d.p.i. Lungs were analyzed on day 21 of infection. (A) Percentage changes in body weights related to initial body weight. (B and C) CFUs of M299 Mtb in lung homogenates and lung weights. (D) Gross macroscopic pathology of the left lung. (E) Total cell counts in the lungs of infected mice. (F–J) Absolute numbers of myeloid cells, dendritic cells, macrophages, monocytes, and neutrophils in naïve and infected WT and *Entpd1*^−/−^ mice (gate strategy in Fig. S3). (K and L) Blinded quantitative histomorphometry of H&E sections showing percentages of inflammation and necrotic areas. (M) Histopathological analysis with Ziehl-Neelsen staining of lung sections. Significant differences between groups are indicated by p < 0.05. Significance levels are represented as follows: p < 0.05 (*), p < 0.01 (**), p < 0.001 (***), and p < 0.0001 (****), determined by the Mann-Whitney non-parametric test or one-way ANOVA with Bonferroni post hoc test. The data represent two independent experiments with three to four mice per group.

The critical role of CD39 in protecting against the development of necrotic lesions and bacterial spread in severe TB may also result from its ability to reduce the eATP sensing by P2RX7. To test this hypothesis, we pharmacologically inhibited P2RX7 signaling *in vivo*. *Entpd1*^−/−^ mice were treated with Brilliant Blue G (BBG), a selective P2RX7 inhibitor with high affinity for P2RX7 relative to other P2 receptors.^37^ The treatment was administered intraperitoneally every two days from day 14–20 p.i. when the mice started to lose weight, as previously reported.^15^ On day 21 p.i., mice treated with the P2RX7 inhibitor showed reduced body weight loss compared to untreated animals (Fig. 7A). In addition, blockade of P2RX7 in WT and *Entpd1*^−/−^ mice increased resistance to Mtb infection as evidenced by reduction of several parameters associated to severe lung pathology, such as bacterial burden, lung weight, the number and size of white nodules and the number of total cells/lung and myeloid cell (for *Entpd1*^−/−^ mice) counts (Fig. 7B–F). Remarkably, P2RX7 inhibition almost completely abolished the differences between infected WT and *Entpd1*^−/−^ mice regarding disease severity. In contrast, the populations of lung dendritic cells and macrophages (for *Entpd1*^−/−^ mice) were increased after BBG treatment (Fig. 7G and H), while a decrease was observed for monocytes (for *Entpd1*^−/−^ mice) and neutrophils (Fig. 7I and J). Furthermore, P2RX7 inhibition resulted in a considerable reduction in pulmonary inflammation and necrosis areas associated with extracellular bacteria (Fig. 7K–M). These results show that the beneficial effects of CD39 in suppressing the development of severe forms of TB in mice are primarily mediated by its ability to prevent eATP sensing by P2RX7.

## Discussion

Necrotic pneumonia is a severe condition associated with pulmonary TB that significantly impacts disease progression and transmission between individuals by promoting pathogen dissemination through lung tissue and the respiratory tract.^38,39^ Despite advances in understanding the role of P2RX7 in the development of severe TB pathology,^13,15,31,40^ important questions regarding the regulation of this pathway remain unanswered. The ability of CD39 to degrade eATP and generate adenosine in the extracellular environment makes this molecule a possible target for regulating P2RX7 activation and the inflammatory response during TB.^41,42^ Addressing this issue, our study reveals a major role for CD39 in TB progression by controlling P2RX7-induced death of myeloid cells, consequently, reducing necrotic lesions and pathogen dissemination in the infected lungs.

The first indication of the involvement of CD39 in the regulation of P2RX7 signaling during TB was the elevated expression of *ENTPD1* and *P2RX7* genes, but not of genes associated with other ecto-ATPases and adenosine signaling (*ENTPD2,3*, *NT5E*, and *ADORA1,2B,3*) in peripheral blood cells of TB patients. Coexpression of genes encoding CD39 and P2RX7 was also observed in NHP granuloma cells, as well as in the blood and lung cells from Mtb-infected C57BL/6 mice. Across different mouse models, the CD39-P2RX7 axis is engaged during active TB. Notably, when comparing resistant C57BL/6 and necrosis-prone C3HeB/FeJ mice under infection conditions associated with severe lung pathology, *Entpd1* expression in peripheral blood was higher in the Mtb-susceptible strain across both H37Rv and HN878 infections, consistent with association with severe disease phenotypes in necrosis-prone experimental models. In bulk lung tissue, *Entpd1* transcriptional levels were also higher in the susceptible C3HeB/FeJ strain and were further increased following infection with the hypervirulent HN878 strain, whereas no difference between Mtb strains was observed in resistant C57BL/6 mice. In contrast, bulk lung *P2rx7* transcriptional mRNA levels increased in C57BL/6 mice infected with HN878 but were reduced in infected C3HeB/FeJ mice. These findings support CD39 and P2RX7 upregulation as biomarkers of active disease, while suggesting that *Entpd1*/CD39 expression in peripheral blood more closely reflects the susceptible severe phenotype. Importantly, in models with extensive necrotic pathology, the original study reported reduced immune-related gene expression signals in lung tissue,^29^ suggesting that transcriptional readouts from late-stage necrotic lesions may underestimate immune cell–associated gene expression in bulk measurements.

Stimuli associated with tissue damage, hypoxia (via hypoxia-inducible factor 1 [HIF1] transcription factor), and inflammation can upregulate CD39 and P2RX7 expression.^16,17,43–47^ Among them, cytokines with systemic activity, such as IL-1β, IL-6, and TNF, as well as type I IFNs, are produced during TB^48^ and may be responsible for the increased expression of CD39 and P2RX7 in peripheral blood cells during active disease.

Other multiple lines of evidence indicate that CD39 restrains TB progression. Experiments with CD39-deficient mice show widespread pulmonary necrosis, increased mycobacterial growth and dissemination in the lung tissue, early body-weight loss, and higher mortality. A neutrophil/monocyte-biased myeloid infiltrate with reciprocal decreases in macrophages and dendritic cells was observed in the absence of CD39, an inflammatory phenotype often tied to worsening of the disease.^30,49,50^ This compositional shift is accompanied by greater caspase-1-associated myeloid cell death susceptibility, suggesting that CD39 protects these cells from undergoing necrosis associated with inflammasome activation, as it has been reported during pyroptosis.^51^ A heightened inflammatory cytokine/chemokine program (IL-1β, IL-6, TNF-α, CXCL1, CCL2) was also observed, indicating that CD39 limits maladaptive myeloid cell activation. In competitive assays, CD39-deficient myeloid cells do not establish in the infected lung compared with wild-type, supporting a model in which CD39 is required cell-intrinsically to sustain myeloid cell fitness and limit pathologic activation.

Notably, pharmacologic P2RX7 blockade attenuated the exaggerated TB phenotype caused by CD39 deficiency, limiting body-weight decline, lowering pulmonary bacterial burden, reducing lung weight and cellularity, and curbing necrotic pneumonia, while reversing the maladaptive myeloid bias (fewer neutrophils/monocytes with relative restoration of macrophages and dendritic cell populations). BBG, the P2RX7 inhibitor used *in vivo*, can hit other targets only at high μM concentrations, but potently and selectively blocks P2RX7.^37^ We administered a regimen widely used in mouse and rat P2X7 studies. Thus, we attribute the observed effects specifically to P2RX7. With treatment, *Entpd1*^−/−^ mice approached wild-type outcomes, suggesting a rescue of disease phenotype. WT lungs lacked necrotic lesions at this stage, whereas vehicle-treated *Entpd1*^−/−^ lungs were extensively necrotic, and P2RX7 inhibition largely abrogated this difference.

Macrophages and endothelial cells of NHP granuloma exhibited high transcriptional levels of genes encoding CD39 and P2RX7. Still, these genes were expressed mainly in the macrophage population, considering their prevalence in this tissue. Concordantly, in the lungs of Mtb-infected WT mice, CD39 and P2RX7 proteins were expressed at higher levels in immune cells than structural cells, particularly in macrophages that showed the highest expression of both molecules within immune cells. Remarkably, the lack of CD39 restricted to the immune cell compartment was sufficient to promote extensive necrotic lesions, together with increased bacterial growth and dissemination in the lung tissue of mice infected with hypervirulent mycobacteria. Although neutrophil influx and death are known contributors to necrotic lesion formation in TB, the reduction of *Entpd1*^−/−^ neutrophils observed in mixed bone marrow chimeras indicates that CD39 loss also impacts this compartment. However, macrophages represent the primary intracellular niche for *M. tuberculosis* and are among the earliest cells infected during disease establishment.^35^ In line with this, our *in vitro* data demonstrate that CD39 deficiency enhances P2RX7-dependent necrotic death of infected macrophages and promotes extracellular bacillary release, both of which are rescued by P2RX7 inhibition. Thus, while neutrophils likely contribute to overall lesion necrosis *in vivo*, our mechanistic focus remains on macrophage death as a proximal driver of pathology in the CD39-deficient context. This reinforces the importance of CD39 expressed in myeloid cells in the regulation of severe lung pathology, but we cannot rule out a role for structural cells, as reciprocal chimeras were not performed. Importantly, a protective effect against severe TB was observed in mice repopulated with immune cells that did not express P2RX7.^40^

It has been reported that macrophages infected with avirulent mycobacteria die through apoptosis, preventing bacterial dissemination to other cells, whereas virulent mycobacteria trigger necrosis of infected macrophages, thereby facilitating pathogen release into the extracellular environment.^11,52,53^ In this context, we have previously shown that P2RX7 signaling is critical in triggering necrotic death of macrophages infected with highly virulent mycobacteria.^13^ Extending these findings, this study demonstrated that CD39 expression on macrophages critically controls the initial trigger for P2RX7-mediated necrotic lesions in pulmonary TB. Our *in vitro* results showed that infected macrophages lacking CD39 were predisposed to necrotic death. Remarkably, P2RX7 inhibition restored macrophage viability and reduced extracellular bacterial release, indicating that CD39 controls these processes in a P2RX7-dependent manner. A parallel finding has been described in sepsis, in which P2RX7 also plays a significant role in disease development.^54^ Interestingly, a similar phenotype was observed in an animal model of sepsis, in which CD39 deficiency promoted P2RX7-triggered cell death, cytokine production, and liver injury.^36^ Importantly, blocking P2RX7 in these animals increased the macrophage population in the lungs of these mice.

CD39 deficiency permits excessive eATP-P2RX7 activation, promoting necrotic death of infected macrophages and contributing to inflammatory amplification and tissue injury *in vivo*. Our data indicate that CD39 acts as a counter-regulatory brake, via eATP hydrolysis, that protects macrophages from necrotic lysis, preserves antimicrobial function, and limits disease progression, highlighting the CD39-P2RX7 axis as a target for host-directed TB therapy.

## Materials and methods

### Mice

Specific pathogen-free C57BL/6 (WT: CD45.2, CD45.1, and heterozygous CD45.1/2) and *Entpd1*^−/−^ (CD45.2) male (6–8-week-old) mice were bred at the isogenic mouse facility at ICB-USP. After infection, mice were maintained in micro-isolator cages with HEPA filters at the Biosafety Laboratory Level 3, FCF, USP. All procedures were performed in accordance with national regulations and ethical guidelines for mouse experimentation (permit no. 3185080318).

### Lethal irradiation and BM reconstitution

Bone marrow cells were harvested from the femur of WT or *Entpd1*^−/−^ mice by flushing with PBS. A single-cell preparation was obtained by carefully cycling through a 26-gauge needle. Recipient CD45.1 mice were irradiated with a dose of 12 Gy from a 137Cs source. After irradiation, 2 × 10^7^ wt, (CD45.2) or *Entpd1*^−/−^ (CD45.2) BM cells were intravenously transferred in 200 μl PBS1x under anesthesia. For mixed BM chimera experiments, a 1:1 mix of 2 × 10^7^ wt, (CD45.1/2) or *Entpd1*^−/−^ (CD45.2) BM cells were intravenously transferred in 200 μl PBS1x under anesthesia to the recipient CD45.1. The chimeric mice were housed for at least 8 weeks before infection and were fed with water containing an antibiotic (0.1 mg/ml of ciprofloxacin) in the first 4 weeks after BM transplantation.

### Mycobacteria and mouse infection

The Mtb strain of the Beijing genotype (strain M299), isolated from a TB patient in the Maputo province, Mozambique, was kindly provided by Dr. Philip Suffys (Oswaldo Cruz Foundation, FIOCRUZ, Rio de Janeiro, Brazil). Frozen aliquots were thawed and grown in Middlebrook 7H9 medium enriched with 10% (vol/vol) ADC (albumin, dextrose, catalase) (Difco, BD Biosciences, USA) and 0.05% (vol/vol) Tween 80 (Sigma-Aldrich) and maintained at 37° C for seven days until mid-log phase in constant agitation. The bacterial suspensions were sonicated in a water bath and vortexed for 1 min to disperse the lumps. The density of bacterial suspensions was determined by using a spectrophotometer at 600 nm. Mice were anesthetized intraperitoneally (i.p.) with ketamine (Vetbrands, Brazil; 100 mg/kg) and xylazine (Vetbrands; 15 mg/kg) and infected intratracheally (i.t.) with ~70–100 (lower dose) or ~150–200 (double dose) bacilli of the highly virulent Mtb Beijing M299 strain.^30^

### P2RX7 inhibition to prevent cell death during lung processing

Several studies report that P2RX7 directly or indirectly inhibits activity and preserves cell viability during collagenase IV digestion of tissue.^55–57^ To prevent cell death during lung digestion and processing to obtain cell suspensions for flow cytometry, infected mice were injected intravenously with brilliant blue G (BBG, Sigma-Aldrich), a P2X receptor inhibitor (45 mg/Kg/mouse in 200 μL of PBS), 30 min before euthanasia (Fig. S4A). The differences in the cell viability between treated and nontreated infected mice before euthanasia are shown in Fig. S4B–G.

### Isolation and counting of lung-infiltrating cells

The dissected lung lobes were washed with sterile PBS 1x, fragmented, and digested with collagenase type IV (0.5 mg/mL, Sigma-Aldrich) and type IV bovine pancreatic DNAse (Roche Diagnostics; 1 mg/mL) in RPMI 1640 medium (Gibco, USA) at 37°C for 40 min under agitation (200 rpm). The remaining lung fragments were mechanically dissociated by passage through a 100 μm pore-size cell strainer and incubated with ACK Lysing Buffer (Thermo Fisher Scientific, USA) at room temperature for one minute to deplete the erythrocytes. Cell suspensions were washed with 10% fetal calf serum (FCS, Gibco) in PBS following centrifugation at 1,200 rpm for 5 min and resuspended in RPMI 1640 medium. Lung cell viability was determined using a trypan blue exclusion assay and a hemocytometer.

### Histopathological analysis

H&E-stained lung sections were scanned as brightfield whole-slide images (WSI) and analyzed in ZEN software (Carl Zeiss). Slides were exported with coded filenames by an investigator not involved in scoring. The analyst was blinded to genotype/treatment/timepoint. Image order was randomized before measurement. Sections with >10% artifact within the ROI were rescanned or excluded before analysis. For each section, the total lung parenchyma was manually outlined as the region of interest (ROI), excluding background glass and obvious artifacts (folds/tears). Large conducting airways and major vessels were excluded from the ROI when clearly demarcated. Areas of granulomatous pneumonia were delineated as fields with dense leukocyte infiltrate replacing the normal alveolar architecture. Within these lesions, necrotic core was defined as acellular/anuclear material with karyor-rhectic debris and/or caseation. ZEN’s area tools were used to record: (i) total lung area, (ii) granulomatous pneumonia area, and (iii) necrotic area. Two prespecified readouts, both normalized to the total lung, were calculated:

$$\text{Pneumonia burden}\;(\%)=\left(\frac{\text{granulomatous pneumonia area}}{\text{total lung area}}\right)\times 100$$

$$\text{Necrosis burden}\;(\%)=\left(\frac{\text{necrotic area}}{\text{total lung area}}\right)\times 100$$

### Phenotypic analysis of lung-infiltrating cells

Isolated lung cells (1 × 10^6^ cells/well) were seeded in round-bottom 96-well plates and stained with Live/dead dye (Thermo Fisher Scientific) to determine cell viability, as described in the datasheet. Lung cells were stained using fluorochrome-labeled monoclonal antibodies to lineage [CD4 (RM4-5), CD8 (S3-6.7), CD19 (1D3) and NK.1 (PK136)], CD11b (M1/70), Ly6G (1A8), Ly6C (AL-21), CD11c (N418), F4/80 (BM-8), CD39 (24DMS1), CD4 (RM4.5), CD8 (S3-6.7), CD44 (IM7) and CD69 (H1.2F3)] (BD Biosciences) for 30 min at 4°C. Cells were fixed with 4% paraformaldehyde for 30 min at 4°C and washed in a staining buffer. Cell acquisition was performed using the LSRFortessa^™^ flow cytometer (BD Biosciences, USA) and FlowJo 10.5.3 software (BD Biosciences). The gating strategy for myeloid cell analysis is shown in Figs. S3–S5.

### Lung macroscopic and microscopic analyses

The harvested lung lobes were washed with sterile PBS 1x and weighed. The lung relative mass was calculated by dividing the mean lung weight in experimental mice by the mean in uninfected controls from their respective groups. The left lung upper lobe was maintained in 10% buffer formalin for 48 h, photographed, and subsequently embedded in paraffin. Histological sections of approximately 4–5 μm were stained using the Hematoxylin and Eosin (H&E) method to visualize tissue alterations, Ziehl Neelsen (ZN) method to detect the presence of acid-fast bacteria (AFB), and Immunofluorescence for TUNEL staining (Thermo Fisher Scientific), as described in the datasheet, to visualize nucleus fragmentation. The samples were examined with an Axioplan microscope (Carl Zeiss Inc., Germany), and the images of lung sections were captured by a Coolpix P995 (Nikon)-coupled device camera.

### Colony-forming unit (CFU) analysis and counting

Corresponding bacterial concentrations in lung homogenate were determined by the colony-forming unit test, using serial 10-fold dilutions of each suspension and plating on Middlebrook 7H10 agar (Difco, Detroit, MI), supplemented with 0.5% glycerol, 10% oleic acid-–albumin-dextrose–catalase enrichment, OADC (BD, Sparks, MD). Plates were cultured at 37°C for 21 days, and total CFU was determined by colony counting.

### BMDM cultures

Murine BMDMs were generated by isolating undifferentiated monocytes from bone marrow from both femurs and tibiae of WT and *Entpd1*^−/−^ mice. Bone marrow was harvested in DMEM/F-12 (Gibco) supplemented with 10% heat-inactivated FBS (Gibco) and flushed through a syringe with a 16-gauge needle. Cells were dispersed with a 5-mL syringe (BD Biosciences) fitted with a 20-gauge needle. Dispersed cells were seeded in culture flasks (T-175) containing 20 mL DMEM/F-12 supplemented with 10% FBS, 25 μg/ml gentamicin (Gibco), and 20% of L929-conditioned media. Cells were incubated at 37°C with 5% CO2, and 20 mL of fresh medium containing L929-conditioned media without gentamicin was added on day 3. On day 7, macrophages were detached by the addition of cold PBS.

### In vitro BMDM infection

Frozen bacterial aliquots were thawed and cultured as described above. Bacterial suspensions were centrifuged at 4,000 rpm for 10 min, resuspended in DMEM/F-12, sonicated for 30 s, and homogenized to reduce bacterial clumping. BMDMs were exposed to M299 Mtb infection at 1:10 MOI. After 3 h, cells were washed three times with room temperature PBS and then cultured in fresh DMEM/F-12 media, supplemented with 3% for 24 h. According to the manufacturer’s instructions, LDH release in the supernatants from BMDM cultures was determined using the CytoTox 96 nonradioactive cytotoxicity assay (Promega). In some experiments, BMDM cells were pre-treated with 10 μM of A740003 (R&D System, #CAT3701/10).

### Statistical analysis

Statistical analyses were performed using the GraphPad Prism 7 software (GraphPad, USA), and differences between groups were considered significant when p < 0.05 (5%). ANOVA and Bonferroni post-test analyzed the simultaneous effects of two factors. One-way ANOVA and Tukey tests were used to evaluate the impact of a single parameter. The Mann-Whitney non-parametric test was used to compare the medians of two independent groups.

### Figure visualization

Figures were organized using Adobe Illustrator 2025 (v.29.2.1) and R (4.4.0), incorporating images from BioRender.com.

## Supplementary Material

MMC1

MMC5

MMC4

MMC3

MMC2

## Appendix A. Supplementary material

Supplementary material to this article can be found online at https://doi.org/10.1016/j.mucimm.2026.03.007.
