# Supplementary figures and images for: CD39 regulates P2RX7-mediated lung necrotic lesions in severe experimental tuberculosis
Source: Mucosal Immunol. Author manuscript; Available in PMC 2026 May 7. (PMC13148415; doi:10.1016/j.mucimm.2026.03.007)

**A**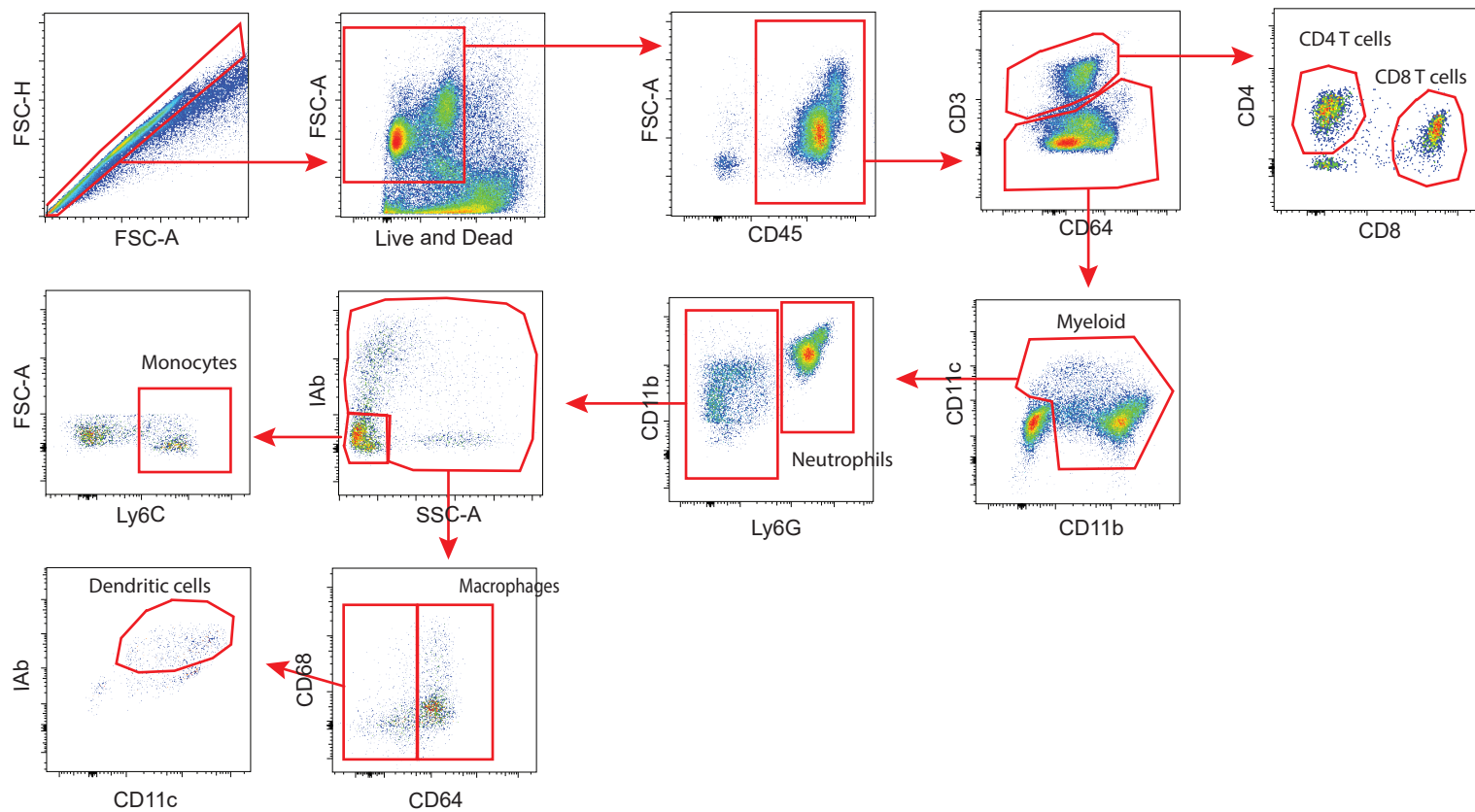**B**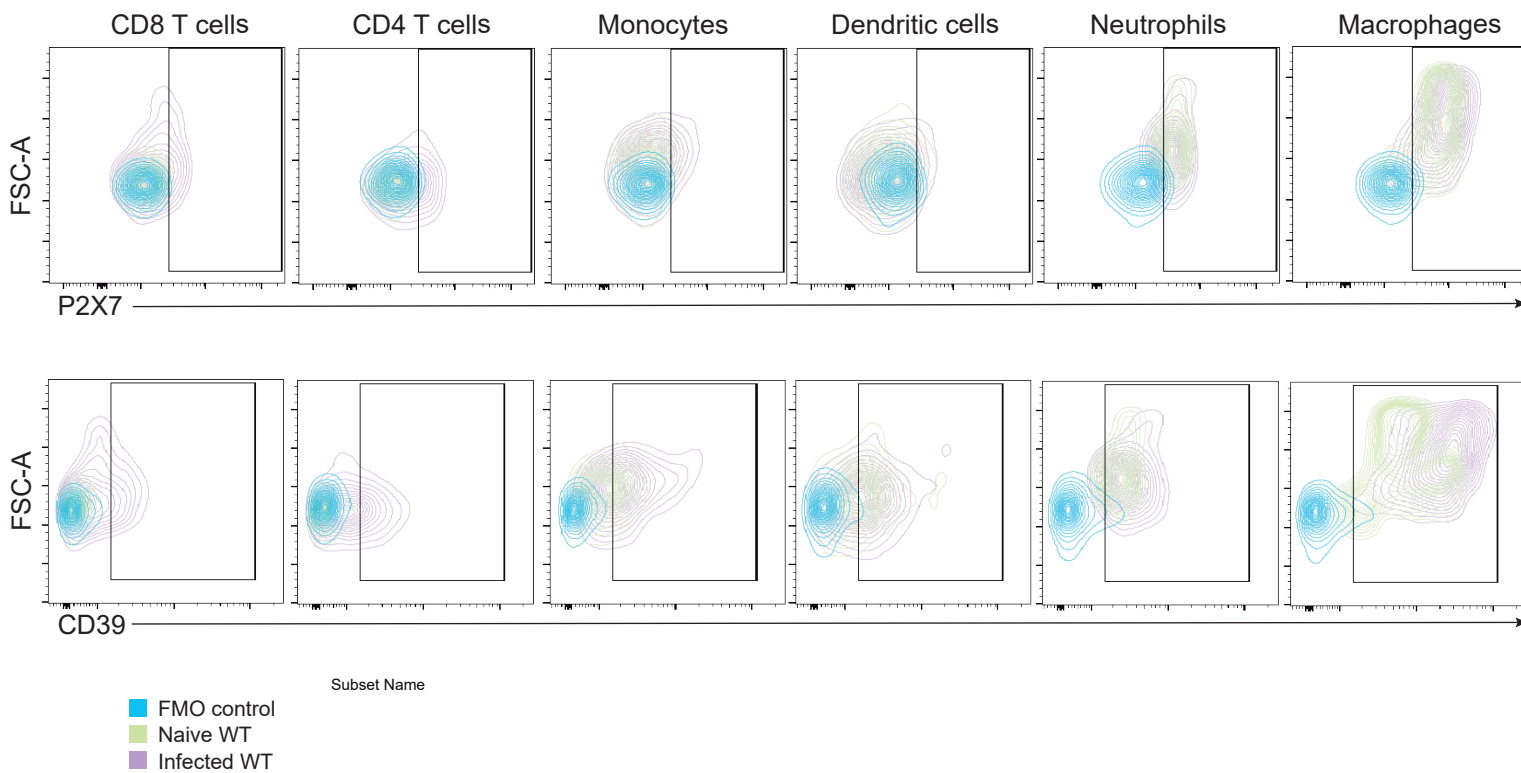

Supplement: MMC1 [file NIHMS2158476-supplement-MMC1.pdf]

**A**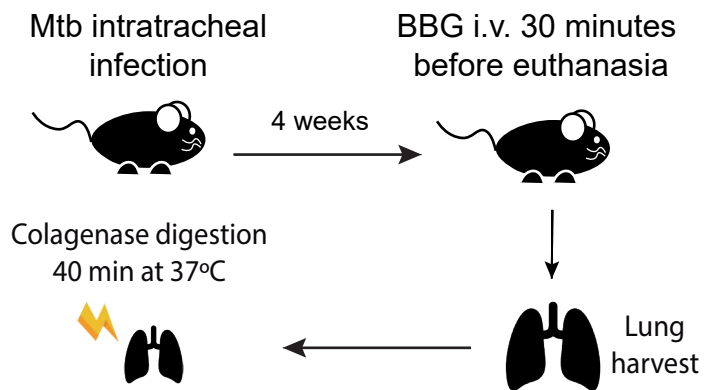**B**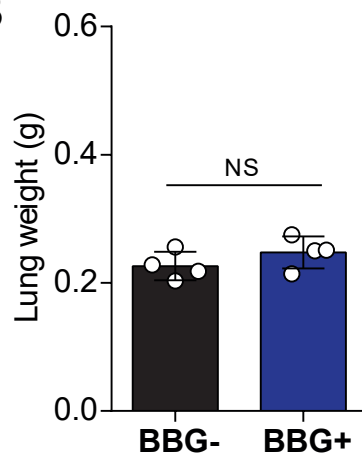**C**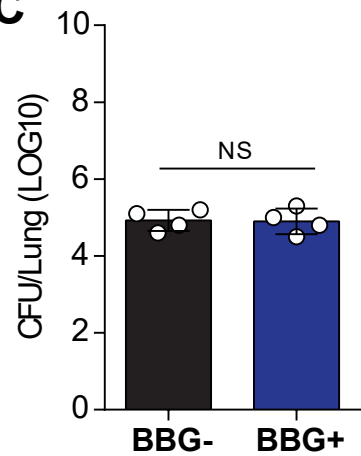**D** Gated on leukocytes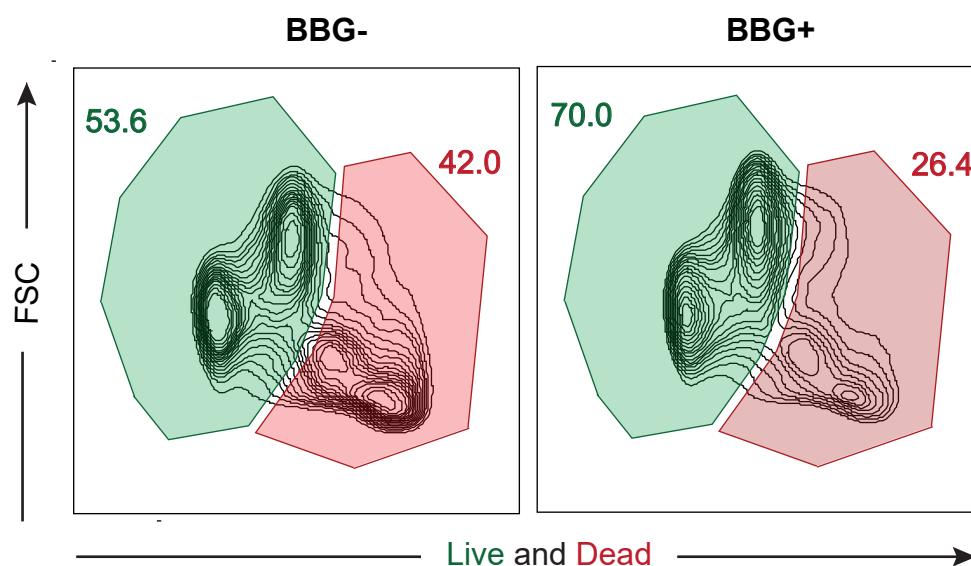**E**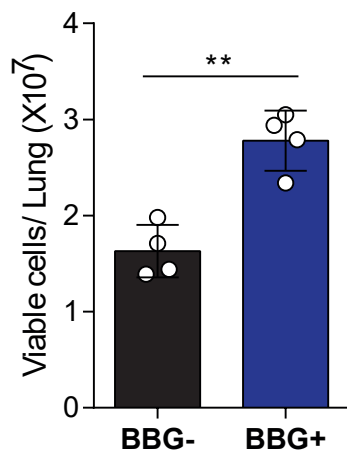**F**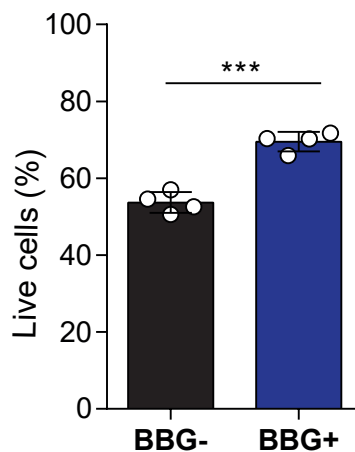**G**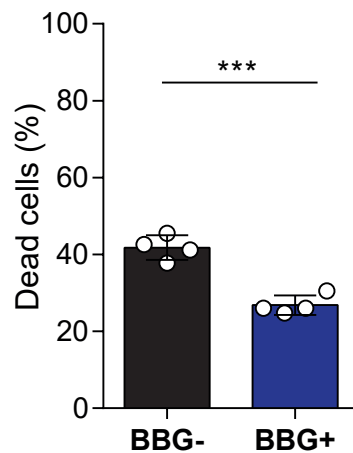

Supplement: MMC4 [file NIHMS2158476-supplement-MMC4.pdf]

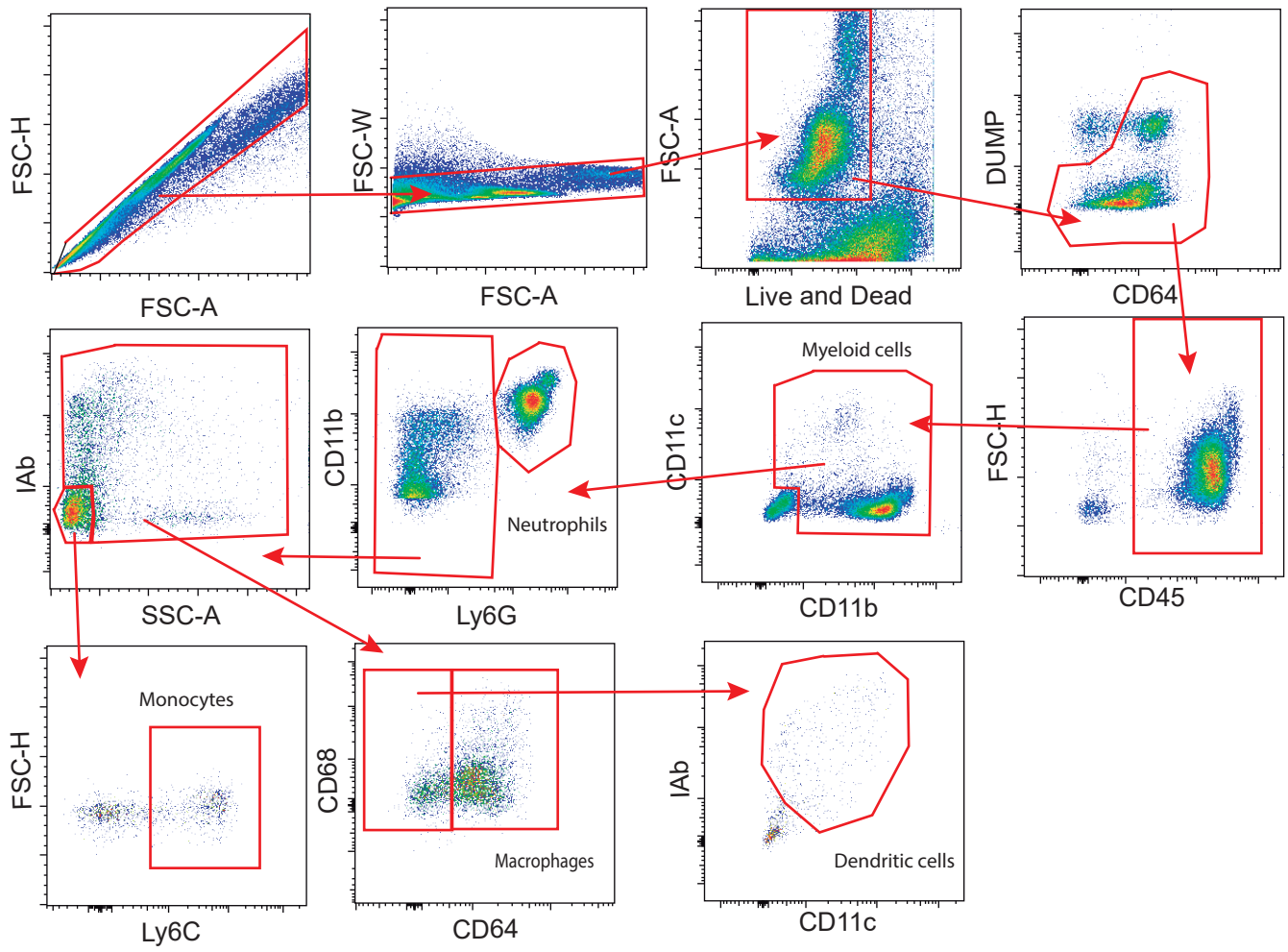

Supplement: MMC3 [file NIHMS2158476-supplement-MMC3.pdf]

**A**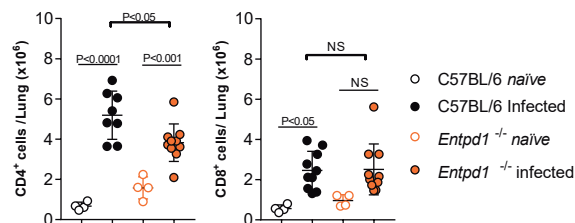**B**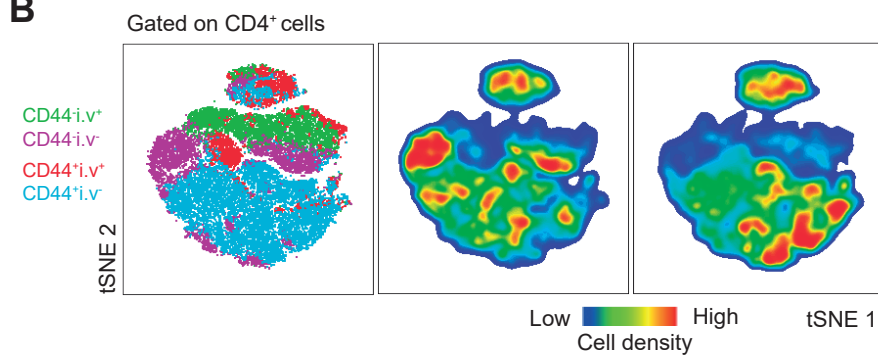**C**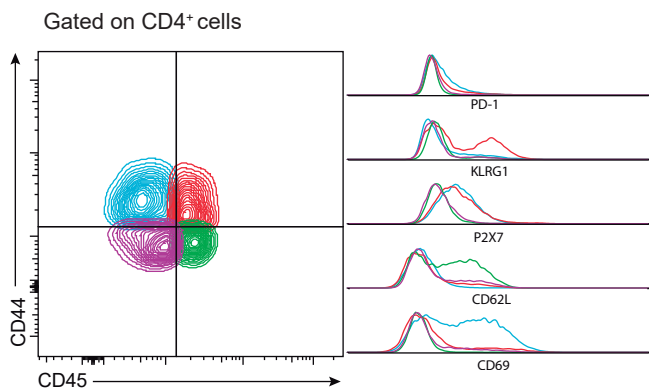**D**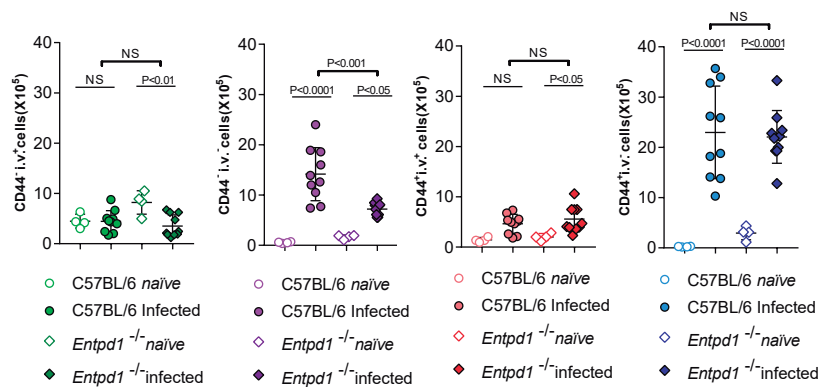**E**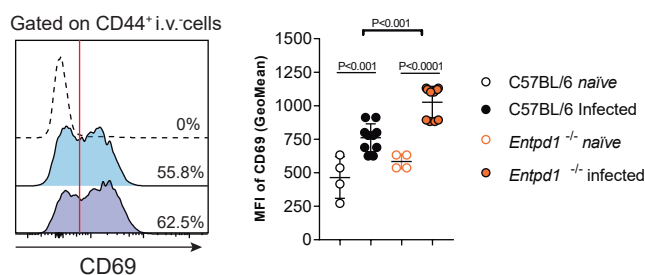**F**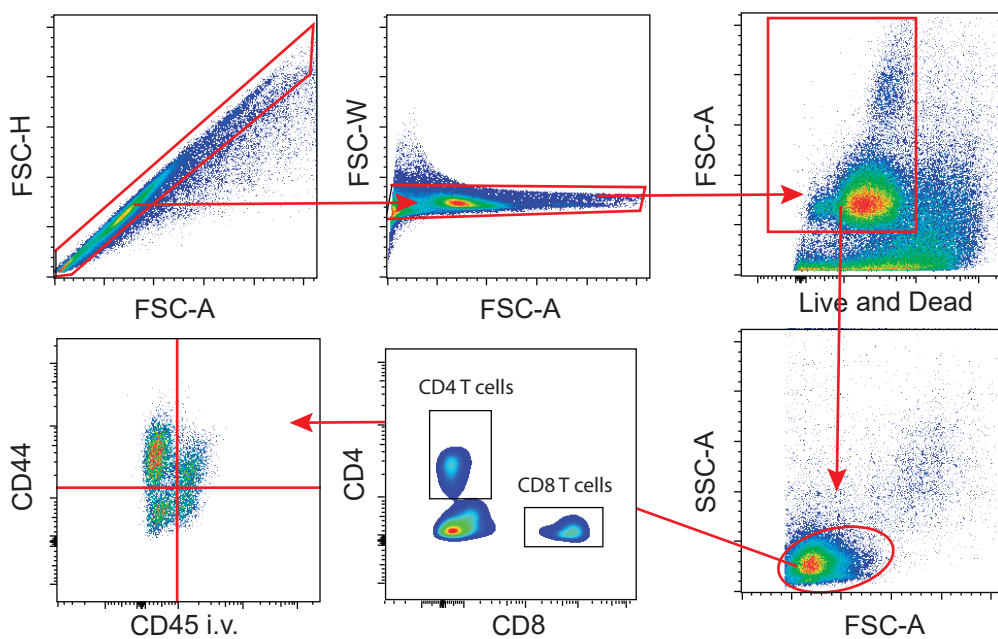

Supplement: MMC2 [file NIHMS2158476-supplement-MMC2.pdf]
