# Supplementary material for: CD39 regulates P2RX7-mediated lung necrotic lesions in severe experimental tuberculosis
Source: Mucosal Immunol. Author manuscript; Available in PMC 2026 May 7. (PMC13148415; doi:10.1016/j.mucimm.2026.03.007)
Supplement: MMC5 [file NIHMS2158476-supplement-MMC5.pdf]

Study accession	Platform Accession	RNA source	Species	Total Samples	Pheno. Classes	Sample size/group	Platform Name	Title	Publication	DOI	Publication date	Contact name
GSE19435	GPL6947	Whole blood	Homo sapiens	33	healthy; active-trested-TB; active-nontreated-TB	12 healthy; 6 active non-treated TB	Illumina HumanHT-12 V3.0 expression beadchip	Transcriptional profiles in Blood of patients with Tuberculosis - Longitudinal Study	An interferon-inducible neutrophil-driven blood transcriptional signature in human tuberculosis	10.1038/nature09247	8/1/2010	Matthew P. R. Berry
GSE19439	GPL6947	Whole blood	Homo sapiens	42	healthy; active-nontreated-TB; latent-nontreated-TB	12 healthy; 13 active non-treated TB	Illumina HumanHT-12 V3.0 expression beadchip	Blood Transcriptional Profiles in Active and Latent Tuberculosis UK (Training Set)	An interferon-inducible neutrophil-driven blood transcriptional signature in human tuberculosis.	10.1038/nature09247	8/1/2010	Matthew P. R. Berry
GSE19444	GPL6947	Whole blood	Homo sapiens	54	healthy; active-nontreated-TB; latent-nontreated-TB	12 healthy; 20 active non-treated TB	Illumina HumanHT-12 V3.0 expression beadchip	Blood Transcriptional Profiles of Active and Latent TB (UK Test Set)	An interferon-inducible neutrophil-driven blood transcriptional signature in human tuberculosis.	10.1038/nature09247	8/1/2010	Matthew P. R. Berry
GSE34608	GPL6480; GPL7731	Whole blood	Homo sapiens	68	healthy; active-nontreated-TB	18 healthy; 8 active non-treated TB	Agilent-014850 Whole Human Genome Microarray 4x44K G4112F; Agilent-019118 Human miRNA Microarray 2.0 G4470B	Gene and microRNA expression in pulmonary tuberculosis and sarcoidosis	Common patterns and disease-related signatures in tuberculosis and sarcoidosis.	10.1073/pnas.1121072109	4/30/2012	Jeroen Maertzdorf
GSE42825	GPL10558	Whole blood	Homo sapiens	42	healthy; active-nontreated-TB; Sarcoidoisis	23 healthy; 8 active non-treated TB	Illumina HumanHT-12 V4.0 expression beadchip	Human whole blood microarray study to compare patients with tuberculosis, sarcoidosis, pneumonia, and lung cancer (validation)	Transcriptional blood signatures distinguish pulmonary tuberculosis, pulmonary sarcoidosis, pneumonias and lung cancers	10.1371/journal.pone.0070630	8/5/2013	Chloe I. Bloom
GSE42826	GPL10558	Whole blood	Homo sapiens	102	healthy; active-nontreated-TB; Sarcoidoisis; Lung cancer	52 healthy; 11 active non-treated TB	Illumina HumanHT-12 V4.0 expression beadchip	Human whole blood microarray study to compare patients with tuberculosis, sarcoidosis, pneumonia, and lung cancer (test)	Transcriptional blood signatures distinguish pulmonary tuberculosis, pulmonary sarcoidosis, pneumonias and lung cancers	10.1371/journal.pone.0070630	8/5/2013	Chloe I. Bloom
GSE42830	GPL10558	Whole blood	Homo sapiens	95	healthy; active-nontreated-TB; Sarcoidoisis; Lung cancer	38 healthy; 16 active non-treated TB	Illumina HumanHT-12 V4.0 expression beadchip	Human whole blood microarray study to compare patients with tuberculosis, sarcoidosis, pneumonia, and lung cancer (training)	Transcriptional blood signatures distinguish pulmonary tuberculosis, pulmonary sarcoidosis, pneumonias and lung cancers	10.1371/journal.pone.0070630	8/5/2013	Chloe I. Bloom
GSE54992	GPL570	PBMC	Homo sapiens	39	healthy; active-nontreated-TB; active-treated-TB; latent-nontreated-TB	6 healthy; 9 active non-treated TB	[HG-U133_Plus_2] Affymetrix Human Genome U133 Plus 2.0 Array	Expression data from peripheral blood	Increased complement C1q level marks active disease in human tuberculosis	10.1371/journal.pone.0092340	3/19/2014	Yi Cai
GSE56153	GPL6883	Whole blood	Homo sapiens	71	healthy; active-nontreated-TB; active-treated-TB; recovered-treated-TB	18 healthy; 18 active non-treated TB	Illumina HumanRef-8 v3.0 expression beadchip	Genome-Wide Expression Profiling Identifies Type 1 Interferon Response Pathways in Active Tuberculosis	Genome-wide expression profiling identifies type 1 interferon response pathways in active tuberculosis	10.1371/journal.pone.0045839	9/21/2012	Tom H. M. Otten
GSE79362	GPL11154	whole blood	Homo sapiens	355	TB progressor; Non-progressor	Adolescent cohort study: 46 progressors, 107 controls; Grand Challenges 6-74 cohort study: 43 progressors, 30 controls	Illumina HiSeq 2000 (Homo sapiens)	A blood RNA signature for tuberculosis disease risk: a prospective cohort study	A blood RNA signature for tuberculosis disease risk: a prospective cohort study	10.1016/S0140-6736(15)01316-1	5/15/2019	Daniel E Zak
GSE107995	GPL20301	whole blood	Homo sapiens	414	Berry_SouthAfrica; Leicester_non_progressor_longitudnal_only; Leicester_with_progressor_longitudinal;	Controls:50; LTBI:49, Active TB:53	Illumina HiSeq 4000 (Homo sapiens)	A modular transcriptional signature identifies phenotypic heterogeneity of human tuberculosis infection	A modular transcriptional signature identifies phenotypic heterogeneity of human tuberculosis infection	10.1038/s41467-018-04579-w	5/15/2019	Akul Singhania
GSE140945	GPL6887, GPL21103	Whole blood; Lung	Mus musculus	224	C57BL/6 naïve; C3HeB/FeJ naïve; C57BL/6 H37RV high; C3HeB/FeJ H37RV high	C57BL/6 uninfected:5; C57BL/6 H37Rv high:5; C3HeBFeJ uninfected:5; C3HeBFeJH37Rv high: 5	Illumina MouseWG-6 v2.0 expression beadchip; Illumina HiSeq 4000 (Mus musculus)	Mouse transcriptome reveals signatures of protection and pathogenesis in human tuberculosis	Mouse transcriptome reveals signatures of protection and pathogenesis in human tuberculosis	10.1038/s41590-020-0610-z	4/9/2020	Anne Ogarra
GSE200151	[illegible]27448; GPL2[illegible]	Lung (Granulomas)	Macaca fascicularis	38	Non- aplicable	26 granulomas from 4 non-human primates at 10 weeks post infection with M. tuberculosis (n = 109,584	NextSeq 550 (Macaca fascicularis); Illumina NovaSeq 6000 (Macaca fascicularis)	Multimodal profiling of lung granulomas in macaques reveals cellular correlates of tuberculosis control	Multimodal profiling of lung granulomas in macaques reveals cellular correlates of tuberculosis control	10.1016/j.immuni.2022.04.004	6/27/2022	Constantine Nicholas Tzouanas
